# Biodiversity protection against anthropogenic climate change: Conservation prioritization of *Castanea sativa* in the South Caucasus based on genetic and ecological metrics

**DOI:** 10.1002/ece3.10068

**Published:** 2023-05-18

**Authors:** Berika Beridze, Katarzyna Sękiewicz, Łukasz Walas, Peter A. Thomas, Irina Danelia, Vahid Fazaliyev, Giorgi Kvartskhava, Jan Sós, Monika Dering

**Affiliations:** ^1^ Institute of Dendrology Polish Academy of Sciences Kórnik Poland; ^2^ Faculty of Biology Adam Mickiewicz University Poznań Poland; ^3^ School of Biological Sciences Keele University Staffordshire UK; ^4^ National Botanical Garden of Georgia Tbilisi Georgia; ^5^ Faculty of Agricultural Science and Bio‐System Engineering Georgian Technical University Tbilisi Georgia; ^6^ Forest Development Service Ministry of Ecology and Natural Resources of Azerbaijan Baku Azerbaijan; ^7^ Department of Silviculture Poznań University of Life Sciences Poznań Poland

**Keywords:** biodiversity, conservation genetics, effective population size, landscape genetics, niche modeling, prioritization, sweet chestnut

## Abstract

The climate drives species distribution and genetic diversity; the latter defines the adaptability of populations and species. The ongoing climate crisis induces tree decline in many regions, compromising the mitigation potential of forests. Scientific‐based strategies for prioritizing forest tree populations are critical to managing the impact of climate change. Identifying future climate refugia, which are locations naturally buffering the negative impact of climate change, may facilitate local conservation. In this work, we conducted the populations' prioritization for *Castanea sativa* (sweet chestnut), a Neogene relict growing in the Caucasus global biodiversity hotspot. We generated genetic and ecological metrics for 21 sites in Georgia and Azerbaijan, which cover the natural range of sweet chestnut across the region. We demonstrated that climate primarily drives the pattern of genetic diversity in *C. sativa*, proved with a significant isolation‐by‐environment model. In future, climate change may significantly reorganize the species' genetic diversity, inducing even some genetic loss, especially in the very distinct eastern fringe of the species range in Azerbaijan. Based on our combined approach, we mapped populations suitable for ex situ and in situ conservation, accounting for genetic variability and the location of future climate refugia.

## Graphical abstract

Studies were conducted in the Caucasus hotspot of biodiversity. Using methods of landscape genetics, we identified the drivers of genetic diversity and differentiation in *Castanea sativa* growing in the isolated Caucasian range. We combined genetic and ecological metrics to conduct conservation prioritization for the species in the region of high conservation importance.

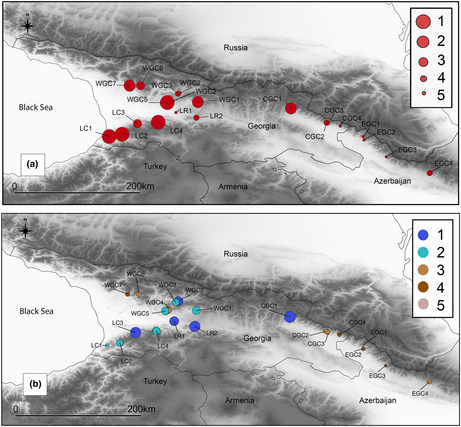

## 1 INTRODUCTION

Beside reduction of CO_2_ emission and decarbonization of energy systems, carbon removal and storage are essential in managing the climate crisis, and studies show that forests are essential carbon sinks (Anderegg et al., 2015; Friedlingstein et al., 2022). Hence, forest‐based natural climate solutions have substantial mitigation potential in the ongoing climate crisis (Griscom et al., 2017, 2019). However, the capability of forests to store carbon depends much on their permanence, which may be compromised by current climate change (Anderegg et al., 2020; Forzieri et al., 2021). Forests are also the most important reservoirs of terrestrial biodiversity (Fraissinet et al., 2023). Thus, their protection is an object within the Aichi Target 11, which aims at preventing biodiversity decline. Despite considerable progress in nature protection (Protected Planet Report, 2020), there is still much to do as biodiversity loss continues and many tree species face the extinction risk (Rivers et al., 2019).

Climate change is leading to the decline of tree populations in many regions (Hartmann et al., 2022). As the climate crisis has become more visible and urgent, practical tools and sound strategies for nature conservation are required (Ali et al., 2021). There is a deep understanding of the role of genetic diversity in climate change mitigation (Hoban et al., 2020; O'Brien et al., 2022; Thurfjell et al., 2022). However, the current climate changes may deprive natural populations of genetic diversity and alter the genetic structure, posing a threat to their resilience and adaptability (Capblancq et al., 2020; Lima et al., 2017; Pauls et al., 2013; Ravenscroft et al., 2015). Consequently, genetic diversity should be a priority in conservation and mitigation plans and yet is still understudied (Heuertz et al., 2023; Hoban et al., 2020).

Genetic patterns are shaped by historical (demographic history) and environmental factors (climate, geographic distance, landscape connectivity), which frequently remain hard to disentangle due to spatial and temporal correlations (Wagner & Fortin, 2013). Their impact can be decoupled and quantified by analytical methods offered by landscape genetics (Orsini et al., 2013; Sękiewicz et al., 2022). By determining the major driving factors of population spatiotemporal genetic diversity and differentiation, we may better inform conservation planning and practice (Hoban et al., 2020). In landscape genetics, the isolation‐by‐distance (IBD) is the baseline model assuming landscape resistance to dispersal resulting in genetic differentiation among populations. However, IBD does not account for the complexity of the landscape. The spatial configuration of landscape elements and habitats is crucial for the spatial distribution of populations and gene flow patterns across the diverse landscape (van Strien et al., 2015). The isolation‐by‐resistance (IBR) model quantifies the impact of different landscape barriers and corridors on gene flow and hence on the genetic structure. Isolation‐by‐colonization (IBC) explains observed genetic patterns by the colonization history of the species including postglacial history. Finally, the isolation‐by‐environment (IBE) model explains differentiation among populations by habitat dissimilarities and ultimately to the selection that drives changes in allele frequencies. Applying landscape genetics tools and models may enable the identification of units within species that are genetically isolated and evolutionarily distinct, and therefore comprise the major units of conservation and management.

Incorporating genetic metrics into the conservation toolbox enables future risks to be defined and conservation actions to be tuned to needs in the extended horizon (Heuertz et al., 2023; Hoban et al., 2021). A host of conservation and mitigation challenges can be met by applying various genetic methods and parameters (Gougherty et al., 2021; Saeki et al., 2018; Walas et al., 2021; Zumwalde et al., 2022). Integrating genetic‐based indices with ecological metrics can make conservation strategies for populations and species more productive (Cheddadi & Khater, 2022). However, in the frame of climate adaptation actions, the approaches for conserving species and communities should also incorporate climate refugia. Future climate refugia may buffer, even if only temporarily, the impacts of the ongoing climate crisis, which may buy species more time needed to evolve (Keppel et al., 2012; Morelli et al., 2016). A protected areas network should include populations with high genetic diversity (adaptability) located in areas with low climate velocity or high overlap with future climates. This, however, challenges the current fixed character of protected areas and needs to be incorporated in future.

To demonstrate the utility of incorporating genetic and ecological information into conservation prioritization, we focused on *Castanea sativa* Mill. (sweet chestnut), a relict Neogene tree species with a long history of domestication around the Mediterranean Basin. The species was extensively cultivated in southern Europe for nuts and timber since the Roman Empire (Fernández‐López et al., 2021; Krebs et al., 2019; Marinoni et al., 2013; Mattioni et al., 2017). Recent studies revealed significant translocation of germplasm between the Iberian and Apennine peninsulas (Fernández‐López et al., 2021) and a considerable gene flow between cultivated orchards and natural populations in the Adriatic region (Tumpa et al., 2022). However, sweet chestnut is also found isolated in the Caucasus ecoregion, a global biodiversity hotspot (Tarkhnishvili et al., 2012; Zazanashvili et al., 2004). The cultivation was rather minor in this part of the species' range, and translocation of germplasm is of low probability (Bobokashvili & Maghradze, 2009). Given this fact, the species distribution range and ecogeographical genetic patterns are supposedly nature states. In the South Caucasus, *C. sativa* naturally grows in Georgia and Azerbaijan. Due to the severe decline caused by the introduced parasitic fungus, *Cryphonectria parasitica* (Murrill) M.E.Barr (Aghayeva & Harrington, 2008; Dumbadze et al., 2018; Tavadze et al., 2013; Wall & Aghayeva, 2014) sweet chestnut was included in the national red lists of both countries. Neither country has active management plans to prevent ongoing population reduction that may lead to genetic erosion. We also lack details about the species' genetic patterns and their underlying factors to plan conservation strategies. A recent study reconstructed the species' demographic history in the South Caucasus and delivered interesting clues about the species' evolutionary history (Beridze et al., 2023). Given the species is facing a significant decline due to fungal disease and that ongoing climate changes may severely impact species inhabiting the region (e.g., Hof & Allen, 2019) conserving and managing sweet chestnut populations in the South Caucasus requires deep recognition of the species' genetic variation and its spatiotemporal attributes.

In this work, we focused on *C. sativa* growing in the South Caucasus in order to shed further light on the utility of an eco‐genetic approach to inform a conservation strategy for this species. We use (i) landscape genetics methods to investigate drivers of genetic diversity and differentiation in *C. sativa* to help assess future risks, (ii) Species Distribution Models (SDMs) to predict future species' distribution and locate areas where climate changes pose the greatest risks across its range in the South Caucasus, (iii) map populations at higher risk of genetic erosion using effective population size (*N*_e_) index, and (iv) use genetic and ecological metrics to prioritize populations for future in situ and ex situ conservation. Considering the region's great climatic variability, we expect to find climatic variables mostly to structure the genetic diversity of the species, according to the IBE model. Previous studies in the region indicated the significant role of a glacial refugium in Colchis (western Georgia) in shaping diversity patterns in trees growing in the region (Beridze et al., 2023; Maharramova et al., 2018; Sękiewicz et al., 2022). Therefore we assume that the highest levels of diversity will be preserved in populations close to the glacial refugia in Colchis, western Georgia; those populations will be most suitable for in situ conservation. We assume that incorporating future climate refugia into conservation management will deliver more precise tips to where locate conservation efforts in order to ensure populations' long‐term persistence and adaptation to a changing climate. However, due to the observed trend of climate deterioration in the region manifesting in lower precipitation and higher temperatures during the last decades, we presume to shrink the species occurrence in the most eastern areas.

## 2 MATERIALS AND METHODS

### 2.1 Genetic data acquisition

**FIGURE 1:**
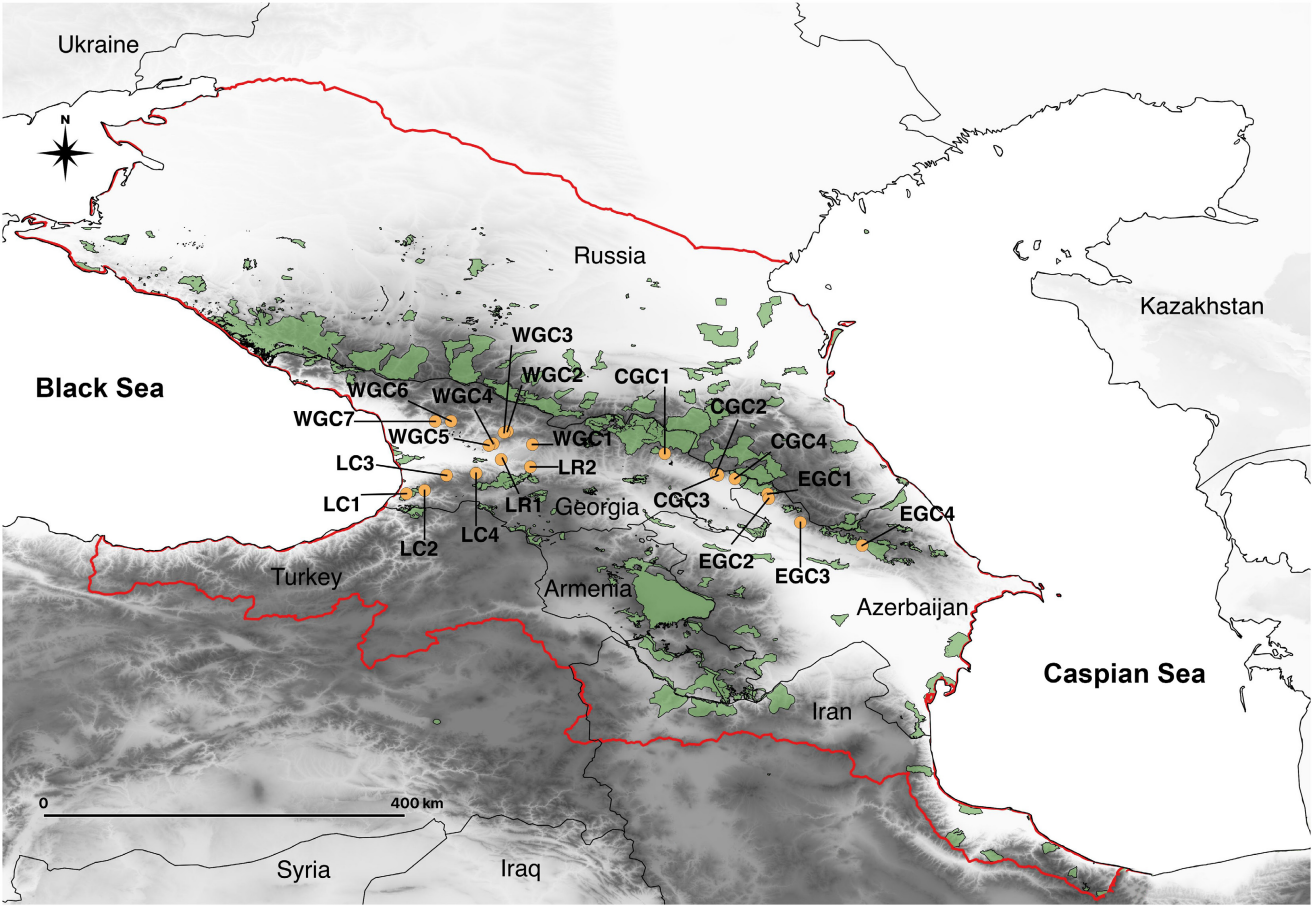
Location of *Castanea sativa* studied populations (see Table 1), boundaries of the Caucasus ecoregion in red, and protected areas of the region are shown as green shapes.

We re‐analyzed the genetic dataset previously generated by Beridze et al. (2023) for landscape genetic analysis. A set of 21 natural populations located in Georgia and Azerbaijan was investigated (Figure 1, Table 1). A total of 626 individuals were genotyped with nine nuclear microsatellites (CsCAT1, CsCAT6, CsCAT14, CsCAT15, CsCAT41, EMCs2, EMCs13, EMCs15, EMCs22) (Buck et al., 2003; Marinoni et al., 2003). Details of the genetic data acquisition are provided in Beridze et al. (2023). For the analyses performed in this study, we extracted the following parameters from Beridze et al. (2023): *Q*‐values at *K* = 4 delivered by STRUCTURE (a membership of individuals to the respective genetic cluster) and the descriptive statistics, such as the number of alleles per locus (*A*), allelic richness (*A*_r_), private alleles (*A*_p_) and a matrix pairwise *F*_ST_ values.

**TABLE 1:** Location of studied populations and conservation index.

Subregion	Pop	Altitude (m a.s.l)	Latitude	Longitude	*N*	*C*_i_
Lesser Caucasus	LC1	187	41.6842	41.8345	30	1.33
	LC2	971	41.7287	42.0781	30	3.11
	LC3	465	41.9293	42.3735	33	4.61
	LC4	582	41.9581	42.7696	31	3.62
Likhi range	LR1	365	42.1429	43.1068	30	4.11
	LR2	899	42.0437	43.4988	30	5.02
Western Greater Caucasus	WGC1	595	42.3442	43.5264	30	3.83
	WGC2	756	42.5205	43.1896	29	4.32
	WGC3	775	42.5038	43.1462	33	3.77
	WGC4	574	42.3557	42.9991	31	2.82
	WGC5	537	42.3329	42.9421	28	3.76
	WGC6	913	42.6569	42.4341	29	2.40
	WGC7	343	42.6548	42.2216	31	1.99
Central Greater Caucasus	CGC1	806	42.2226	45.3037	18	4.66
	CGC2	679	41.9472	45.9865	30	2.21
	CGC3	559	41.9326	46.0194	27	2.48
	CGC4	691	41.8854	46.2457	22	1.76
Eastern Greater Caucasus	EGC1	963	41.6796	46.6864	33	0.97
	EGC2	670	41.6194	46.6990	31	1.65
	EGC3	852	41.2976	47.1249	29	1.18
	EGC4	1058	40.9883	47.9544	41	2.01

Abbreviations: *C*_i_, conservation index; *N*, number of individuals.

### 2.2 Landscape genetic analyses

We performed a series of distance‐based redundancy analyses (dbRDA) to assess the relative role of the landscape variables (orography—*oro*., geographic location—*geo*., climate—*clim*., and species genetic ancestry—*anc*.) in structuring the neutral genetic variation in *C. sativa* in the South Caucasus. The analysis was applied to untangle confounded and constrained contributions of the above explanatory variables on a variation in dissimilarity matrices. It explores the linear relationship between the dissimilarity matrix and explanatory variables and measures in a nonlinear relationship (e.g., geography, climate). As a response matrix, we used Slatkin's linearized pairwise *F*_ST_ values (*F*_ST_/1−*F*_ST_).

To assess the impact of the climate (*clim*.) on the observed differentiation pattern, we tested for IBE. For each locality, 19 bioclimatic variables for the current period were downloaded from CHELSA (1981–2010, Karger et al., 2017), while annual potential evapotranspiration (annualPET), aridity index (aridityIndexThornthwaite), relative wetness and aridity (climaticMoistureIndex) were taken from ENVIREM (Title & Bemmels, 2018). All climatic variables were standardized using *scale* R functions (R Core Team, 2022). To maximize the variance explained by a set of climatic predictors and to avoid overfitting and collinearity in the dbRDA models, we applied the forward selection procedure using the *ordiR2step* function in the vegan R package (Oksanen et al., 2020) following variable significance of *p* < .01 and the adjusted *R* ^2^ of the global model using 9999 permutations.

To analyze the possible influence of the pairwise geographic distance (*geo*.) on the genetic differentiation of populations, we tested the IBD model using the coordinates of the population (latitude and longitude) as a proxy. However, since IBD considers only the “flat landscape,” an unrealistic assumption in the rugged topography of the South Caucasus, we tested the impact of orographic complexity (*oro*.) in the frame of the IBR model based on circuit theory (McRae & Beier, 2007). For this purpose, the terrain ruggedness index (TRI) calculated in QGIS 3.16.3 “Białowieża” (QGIS Development Team, 2022) was used. To generate the vector from the dissimilarity matrix of orographic complexity, we applied the principal component of neighbor matrices (PCNM) procedure (Borcard & Legendre, 2002) using the *pcnm* function in the *vegan* R package. The first score components were retained in downstream analyses.

As the final factor possibly governing the pattern of diversity and differentiation, the impact of historical demographic events (divergence and admixture) was considered. Here, we used the STRUCTURE results previously performed for studied populations by Beridze et al. (2023). Specifically, *Q*‐membership values at *K* = 4, which defined the four evolutionary lineages in the contemporary gene pool of sweet chestnut, were extracted to test the IBC and trace the ancestry factor in the genetic diversity pattern. We used population scores along with the first two principal components (anc1, and anc2) from principal component analysis (PCA) performed on the set of *Q*‐membership values using the *prcomp* function in R (R Core Team, 2022).

All predictors included in the final models were scaled using the *scale* function in R, and the correlation between them was assessed using the *cor* function (R Core Team, 2022). Finally, the dbRDA analyses were run using the *capscale* function implemented in the vegan R package (Oksanen et al., 2020). The significance of the models was tested using the “*anova*” function based on 9999 permutations.

### 2.3 Effective population size and bottleneck effect

We chose two existing methods of estimating *N*_e_ and their specificity, namely NeEstimator v.2.01 (Do et al., 2014) and Migrate‐n v. 3.7.2 (Beerli et al., 2019). Both methods are based on a single‐sample approach but differ in terms of the methodology and timescale included. The contemporary *N*_eLD_ in NeEstimator is assessed using the linkage disequilibrium (LD) method and refers to the recent past, a few generations back in time. In NeEstimator, a random‐mating model was chosen for analysis; low‐frequency alleles (≤0.02) were excluded from the procedure. In contrast, Migrate‐n computes *N*_eCOAL_ using the Bayesian inference method based on the coalescence theory (Beerli et al., 2019), and it refers to the harmonic mean of *N*_e_ changing over the whole genealogy. This software estimates *N*_e_ as the mutation‐scaled parameter, which is Ө = 4*N*_e_ *μ*, for diploid nuclear organisms, where *μ* is the mutation rate. Ө was converted into *N*_e_ based on the mutation rate of 5.47 × 10^−5^ estimated using the set of SSRs by Beridze et al. (2023). In Migrate‐n, we used the Brownian motion model, with starting conditions based on *F*_ST_ and uniform prior distribution, to estimate theta Ө with minimum, maximum, and delta values of 0, 100, and 10, respectively. The process included 10^7^ iterations with a burn‐in of 1000. The static heating scheme was used with four chains at different temperatures, that is, 1.0, 1.5, 3.0, and 100,000.0.

The M‐ratio method (Garza & Williamson, 2001) implemented in INEst v. 2.2 (Chybicki & Burczyk, 2008) was used to infer the possibility of recent bottlenecks. Analyses were done by choosing a two‐phase model (TPM) with default parameters. The Wilcoxon signed‐rank test was chosen to verify the significance of the deficiency in the M‐ratio based on 10^6^ coalescent simulations.

### 2.4 Species niche modeling and changes in genetic structure

We investigated future range shifts in the distribution of *C. sativa* using the maximum entropy algorithm implemented in MaxEnt 3.4.3 (Phillips et al., 2004). The latest available bioclimatic data from the Coupled Model Intercomparison Project (CMIP6) were used (Eyring et al., 2016). Three models, GFDL‐ESM4, MPI‐ESM1‐2‐HR, and UKESM1‐0‐LL out of five currently available in CHELSA 1.2 (Karger et al., 2017) were chosen based on suggestions of Karger et al. (2023). To deliver results on the feasible future climate change scenarios, we used two shared socioeconomic pathways (ssp) incorporated into the latest Coupled Model Intercomparison Project (CMIP6) instead of representative concentration pathways (RCPs, CMIP5) (Riahi et al., 2017). Specifically, we used more realistic ssp370 and more severe ssp585 scenarios to deliver predictions based on two relatively extreme and likely versions of the climatic future (Hausfather & Peters, 2020). Bioclimatic variables (bio1‐19) available on the CHELSA were downloaded for 2041–2070 and 2071–2100 and current (1981–2010) periods at 30 arc‐sec resolution. To avoid autocorrelation in the landscape between bioclimatic variables, the *vif* function implemented in the *usdm* R package (Naimi et al., 2014) and the *layerStats* function from package Raster 3.5 to 15 (Hijmans et al., 2015) were used. Variables with large VIF values (>10) were excluded one by one using a stepwise procedure. As a result, eight bioclimatic variables, bio1 (annual mean temperature), bio3 (isothermality), bio4 (temperature seasonality), bio8 (mean temperature of the wettest quarter), bio9 (mean temperature of the driest quarter), bio15 (precipitation seasonality), bio18 (precipitation of the warmest quarter) and bio19 (precipitation of the coldest quarter), were used in the subsequent modeling procedure. A total of 88 georeferenced spots of *C. sativa* occurrence were taken from Beridze et al. (2023). Occurrence data were manually checked for duplications. MaxEnt was run with 100 replicates using bootstrap resampling, the maximum number of iterations was set at 10^4^, and the convergence threshold was set at 10^−5^ with the logistic output of the model prediction for suitability. The maximum number of background points was set to 10,000 and regularization multiplier to 1. The “random seed” option was applied to validate the models, where 20% of the occurrence points were random sampling as test data, the remaining points were used as training data, and a random test partition was used for each run. Finally, the Area Under the Curve (AUC) and True Skill Statistics (TSS) were used to evaluate each model's accuracy (Allouche et al., 2006; Ancillotto et al., 2019; Soilhi et al., 2022). We applied to the results a baseline threshold of higher or equal to 15% and 70% of habitat suitability to deliver figures and calculate distribution areas in QGIS. Additionally, we calculated the mean altitudinal range shift according to the respective climatic scenario in SAGA‐GIS 8.0.1 (Conrad et al., 2015).

Principal Component Analysis was used to explore the climatic distinctiveness of sweet chestnut populations growing in the West and East South Caucasus. The analysis was performed on bioclimatic variables that showed low multicollinearity in the studied region. The analysis was prepared using the *prcomp* function in R. The final graph was delivered in the *ggbiplot* R package with scale factor (*obs.scale*) set to 2 to draw the first two PCs. Also, we explored the species autecology by comparing bioclimatic and environmental variables. For this analysis, we divided the species' natural range into six distributional domains: Western and Eastern Caucasus, Anatolia, and Europe's three main Peninsulas (Iberian, Apennine, and Balkan). We collected the natural georeferenced occurrence of sweet chestnut (7173 spots in total) from the GBIF database (GBIF.org, April 24, 2022, https://doi.org/10.15468/dl.h22kfz) based on the available data on the species' natural range (Caudullo et al., 2017). The ecoplots were built using bio18, bio19, aridityIndexThornthwaite, and climaticMoistureIndex.

Finally, to explore the potential changes in population genetic structure in response to the climate change scenarios, we employed POPS 1.2 (Jay et al., 2015). The software uses the Bayesian clustering methods on genetic data associated with geographic and delivered environmental variables. We applied the admixture model and four bioclimatic variables that showed the highest contribution in SDMs (see Section 3). Thus, for modeling the 2071–2100 period in scenarios ssp370 and ssp585, we used annual mean temperature (bio1), isothermality (bio3), precipitation of the warmest quarter (bio18), and precipitation of the coldest quarter (bio19) retrieved from CHELSA. Three climatic models were used for the analyses: GFDL‐ESM4, MPI‐ESM1‐2‐HR, and ESM1‐0‐LL (Karger et al., 2017). The software was run with 10^5^ seeps, 10^4^ burn‐in, and 10 replications for each *K* (number of clusters). The tested *K* varied from 2 to 5 and was chosen based on the results of STRUCTURE's generic clustering performed by Beridze et al. (2023). Optimal *K* and best run were chosen according to DIC value and correlation plot (Appendix S1, Figure S4). Next, the output was averaged between the three climatic models used (see the results in Appendix S2, Figure S1–S3). For final visualization in QGIS, we overlaid POPS results on the hypothetical distribution area of the species for each corresponding scenario delivered in SDM (ssp370, ssp858; threshold applied—15% of occurrence).

### 2.5 Conservation prioritization

To assess the conservation priority among studied populations, we applied two approaches. First, we used a relatively convenient method of Reserve Selection based on allelic diversity metrics implemented in DIVA‐GIS 2.3.2 (Hijmans et al., 2001). This software identifies a minimum number of populations across the landscape representing all allelic diversity found in the data. The analysis was done with an equal weight option using a maximum iteration of 100; the result was delivered using QGIS.

Secondly, we adopted the conservation index (*C*_i_) developed by (Cheddadi et al., 2022), which integrates genetic and environmental proxies of conservation priority. This approach refers to the idea of climate change refugia, that is, the areas that may support populations thriving in a changing climate. Initially, the authors used allelic richness, ruggedness index, and climatic data to calculate *C*_i_ for *Cedrus atlantica*, a high‐mountain species of North Africa. Since *C. sativa* occurs in lower altitudes and displays a different set of life history traits, we modified the original formula. The topographic complexity was replaced by the forest cover percentage estimated in a polygon having a radius of 20 km for each studied population, which served as a surrogate of the forest continuity. *Castanea sativa* is an insect‐pollinated species, so the connectivity of populations (e.g., forest cover continuity) might be a crucial factor in pollinators' movement and gene flow. The values of forest cover data in the Caucasus were gained from Buchner et al. (2020). Specifically, the formula was:

$$C_{i}=\frac{\left(A_{r}\times F_{c}\right)}{C_{l}}$$

where *A*_r_ denotes allelic richness, *F*_c_ is forest percentage from each population's polygon, and *C*_l_ is a geometric mean between the bioclimatic variables of the current and future (2071–2100) period according to the scenario ssp585. We used precipitation of the warmest quarter (bio18) and precipitation of the coldest quarter (bio19) to calculate *C*_i_ as they showed the highest contribution to SDMs (see the Section 3), implying the highest importance of these two variables for the species niche in the studied region. Higher values of *A*_r_ and *F*_c_ act toward increasing the conservation priority for a given population, while the higher shift between the current and the future climatic decreases the conservation priority due to a higher risk of habitat instability caused by climate changes.

## 3 RESULTS

### 3.1 Allelic diversity

Details of the genetic diversity of populations can be found in Beridze et al. (2023). The average number of alleles per population ranged from 3.56 (EGC3, CGC4) to 6.67 (LC1, LC4), with a mean of 5.25. The mean number for *A*_r_ was 4.63, with values ranging from 3.20 in EGC3 to 5.86 in LC1. The eastern populations (CGC and EGC) displayed significantly lower allelic diversity (*p* < .05) in comparison to western‐located stands (LC and WGC). There were no private alleles in more than half of the populations studied, and the distribution of *A*_p_ displayed an apparent west–east gradient with the highest number of private alleles in the western range.

### 3.2 Landscape genetics

The correlation values among explanatory variables used in the dbRDA analysis can be viewed in supplementary materials (Appendix S1, Figure S1). Only two bioclimatic variables (precipitation seasonality and aridity index) out of the 22 applied were identified as significantly associated with genetic variation and used in subsequent IBE model. The projection of populations and environmental variables along the first two dbRDA axes explained only 24.2% of the total variance (Appendix S1, Figure S2). Accordingly, CAP1 was positively loaded with the aridity index. In contrast, CAP2 was positively loaded with bio15. Populations from the eastern part of the range (CGC2‐3, EGC1‐3) diverged from western populations growing in more humid habitats, with precipitation relatively equally distributed annually.

The full model I, which incorporated all explanatory variables (*clim*., *geo*., *oro*., and *anc*.) explained 60% of the total variance, showing a strong statistically significant association (adj*R* ^2^ = .864, *p* < .01) (Table 2). The partial dbRDAs performed to decompose their contribution to among‐population variation showed that the IBR incorporating orography (*oro*.) was not significant (*p* > .05, Table 2), and thus this variable was excluded from further analyses. Together, climate (*clim*.), neutral genetic structure (*anc*.), and geography (*geo*.) significantly explained 54% of the total variance. However, significant variation was attributed exclusively to the pure effect of geography (20%; *p* < .05) and climate (19%; *p* < .05).

**TABLE 2:** The effect of limate (*clim*.), geographic (*geo*.), orographic (*oro*.), and neutral genetic structure (ancestry; *anc*.) to among‐population differentiation (Slatkin's *Fst*) reveald by partial dbRDA for C. sativa populations in the South Caucasus.

Model	adj*R* ^2^	*p* (>*F*)	Proportion of explained variance	Proportion of unexplained variance	Proportion of confounded variance
Full model I: F_ST_ ~ clim. + anc. + oro. + geo.	.864	.003**	0.597	0.404	—
Pure geography (IBD): F_ST_ ~ geo. |(clim. + anc. + oro.)	**.445**	.0105*	**0.227**	**0.404**	**0.369**
Pure ancestry (IBC): F_ST_ ~ anc. |(clim. + oro. + geo.)	**.326**	.0318*	**0.169**	**0.404**	**0.428**
Pure climat (IBE): F_ST_ ~ clim.|(anc. + oro. + geo.)	**.406**	.0197*	**0.208**	**0.404**	**0.388**
Pure orography (IBR_oro.): F_ST_ ~ oro.|(clim. + anc. + geo.)	.097	.197	0.049	0.404	0.548
Total unexplained	0.404				
Total explained	0.653				
Full model II: F_ST_ ~ *clim*. + *anc*. + *geo*.	.768	.0035**	0.548	0.452	—
Pure geography (IBD): F_ST_ ~ *geo*.|(*clim*. + *anc*.)	**.362**	.0274*	**0.204**	**0.452**	**0.343**
Pure ancestry (IBC): F_ST_ ~ *anc*.|(*clim*. + *geo*.)	.251	.084	0.147	0.452	0.401
Pure climate (IBE): F_ST_ ~ *clim*.|(*anc*. + *geo*.)	**.330**	.0374*	**0.188**	**0.452**	**0.360**
Total unexplained	0.452				
Total explained	0.539				
Full model III: F_ST_ ~ geo. + *clim*.	.516	.0041**	**0.4011**	0.5989	—
Pure geography (IBD): F_ST_ ~ geo.|(*clim*.)	.2156	.0532	0.1586	0.5989	0.2425
Pure climate (IBE): F_ST_ ~ *clim*.|(*geo*.)	**.269**	.0332*	**0.1903**	**0.5989**	**0.211**
Total unexplained	0.5989				
Total explained	0.3489				

*Note*: IBD—isolation‐by‐distance, variable *geo*. longitude and latitude; IBC—isolation‐by‐colonization, variable *anc*. ancestral values of divergence and admixture; IBE—isolation‐by‐environment, variable *clim*. climatic data; IBR—isolation‐by‐resistance, variable *oro*. tomographic complexity (terrain ruggedness index). The statistically significant values are bolded.

*p < .05; **p < .01.

Finally, after excluding the nonsignificant effect of ancestry (14% *p* = .084), the effect of climate was highly significant when controlling for geography and explained 19% of total genetic variation (40% of the variation explained by the full model III), while geography itself was not significant (16%; *p* = .0532; Table 2). The largest proportion of genetic variance could not be uniquely attributed to any of the tested explanatory variables (56% of explained variation).

### 3.3 Effective population size and bottleneck effect

The current effective population size (*N*_eLD_) varied widely from 25.6 in CGC1 to 2718.6 in WGC3 (Table 3). Generally, populations from the western distributional area had a higher effective population size than the stands in the eastern margins. Theta delivered by Migrate‐n ranged from 0.16 in WGC5 to 1.28 in WGC2 and roughly repeated the same spatial pattern. Transforming Ө into population size with the mutation rate of 5.47 × 10^−5^ gave values much higher than *N*_eLD_. The highest value of *N*_eLD_ was reported in WGC2 (93602) and the lowest in WGC5 (11700). In the majority of populations, we noted signs of a bottleneck (*p* < .05); the exceptions were WGC6, WGC7, and CGC1 (Table 3).

**TABLE 3:** Estimations of the effective population size (*Ne*_LD_ and *Ne*_COAL_) and recent bottlenecks in the studied populations of *C. sativa* in the South Caucasus.

Pop	*N*_eLD_	95% CI	Theta (Ө)	95% CI	*N*_eCOAL_	95% CI	MR	MReq	*p*‐value
LC1	114.2	42.0–∞	1.19	0.0–2.33	87,020	0.0–170,384	0.601	0.800	**.0097**
LC2	195.0	46.7–∞	0.97	0.0–2.53	70,932	0.0–185,009	0.561	0.813	**.0038**
LC3	41.2	25.0–86.5	0.72	0.0–1.60	52,651	0.0–117,002	0.647	0.811	**.0276**
LC4	47.4	26.2–131.8	0.85	0.0–2.47	62,157	0.0–180,622	0.611	0.808	**.0277**
LR1	−1791.0	71.2–∞	1.18	0.0–2.93	86,289	0.0–214,260	0.580	0.826	**.0038**
LR2	80.3	30.6–∞	0.63	0.0–2.20	46,070	0.0–160,878	0.582	0.844	**.0044**
WGC1	46.9	21.1–496.9	0.58	0.0–2.40	42,413	0.0–175,503	0.603	0.844	**.0397**
WGC2	76.6	26.9–∞	1.28	0.0–2.67	93,602	0.0–195,247	0.557	0.843	**.0198**
WGC3	2718.6	73.6–∞	0.80	0.0–2.80	58,501	0.0–204,753	0.595	0.839	**.0273**
WGC4	57.7	25.2–1313.4	0.86	0.0–2.47	62,888	0.0–180,622	0.561	0.853	**.0040**
WGC5	−259.6	97.6–∞	0.16	0.0–2.47	11,700	0.0–180,622	0.518	0.807	**.0001**
WGC6	38.8	19.5–153.1	1.12	0.0–2.73	81,901	0.0–199,634	0.635	0.827	**.0750**
WGC7	71.5	29.7–∞	0.84	0.0–2.40	61,426	0.0–175,503	0.708	0.827	.1262
CGC1	25.6	11.8–144.9	0.70	0.0–2.60	51,188	0.0–190,128	0.573	0.830	.0823
CGC2	45.5	19.5–1042.7	0.38	0.0–2.80	27,788	0.0–204,753	0.531	0.852	**.0042**
CGC3	28.4	15.0–81.3	0.70	0.0–1.87	51,188	0.0–136,746	0.570	0.832	**.0117**
CGC4	72.2	16.6–∞	0.76	0.0–2.33	55,576	0.0–170,384	0.624	0.875	**.0117**
EGC1	39.3	20.1–128.2	0.89	0.0–2.53	65,082	0.0–185,009	0.520	0.851	**.0001**
EGC2	28.4	14.8–76.7	0.77	0.0–2.40	56,307	0.0–175,503	0.526	0.866	**.0121**
EGC3	102.7	22.6–∞	0.86	0.0–2.00	62,889	0.0–146,252	0.451	0.880	**.0040**
EGC4	50.0	22.5–312.9	0.44	0.0–2.53	32,176	0.0–185,009	0.518	0.882	**.0039**

*Note*: *N*_eLD_—effective population size with parametric 95% of the confidence interval; Theta—parameter with 95% of CI; *N*_eCOAL_—calculated using Ө and mutation rate (5.47 × 10^−5^) and 95% CI; *MR*—the mean observed M‐ratio; MReq—M‐ratio generated under mutation‐drift equilibrium and *p*‐value of Wilcoxon signed‐rank test significance. The statistically significant values are bolded.

### 3.4 Current and future species range based on climatic variables and genetic structure

For all SDMs (Table 4), high AUC and TSS values were obtained, implying the reliability of the analyses. The precipitation of the coldest quarter (bio19, 37.8%), precipitation of the warmest quarter (bio18, 21.9%), annual mean temperature (bio1; 20.4%), and isothermality (bio3, 10.4%) had the highest contribution in all tested models (Appendix S2, Table S2).

**TABLE 4:** The Area Under the Curve (AUC) and True Skill Statistics (TSS) values, future distributional areas, and altitudinal distribution for current and future periods (averaged across the models).

Model	Time interval	Change scenario	AUC	Distrib. area (≥15%)	Change in distribution^a^ (≥70%)	Distrib. area (≥70%)	Change in distribution^a^ (≥70%)	Altitudinal distribution min.–max.
			TSS					
Current	1981–2010	N/A	0.970	79,935	N/A	13,538	N/A	1231–1350
			0.809					
Average of Future 1, Future 2, and Future 3	2041–2070	ssp370	0.972	85,602	+5667	11,313	−2225	1524–1666
			0.814					
		ssp585	0.970	92,886	+12,951	10,379	−3159	1538–1684
			0.813					
	2071–2100	ssp370	0.971	87,476	+7541	7527	−6011	1641–1810
			0.809					
		ssp585	0.972	88,954	+9019	4220	−9318	1630–1783
			0.811					

*Note*: Total area of distribution (in km^2^) and mean altitudinal range were calculated in QGIS and SAGA by applying a 15% threshold to hypothetical distributional occurrences of species delivered by MAXENT. Danielson and Gesch (2011) data were used to calculate the altitudinal range. Future 1—GFDL‐ESM4, Future 2—MPI‐ESM1‐2‐HR, and Future 3—UKESM1‐0‐L.

^a^ Change in area distribution are calculated by subtracting distribution of particular model to the current occurrence of the species.

**FIGURE 2:**
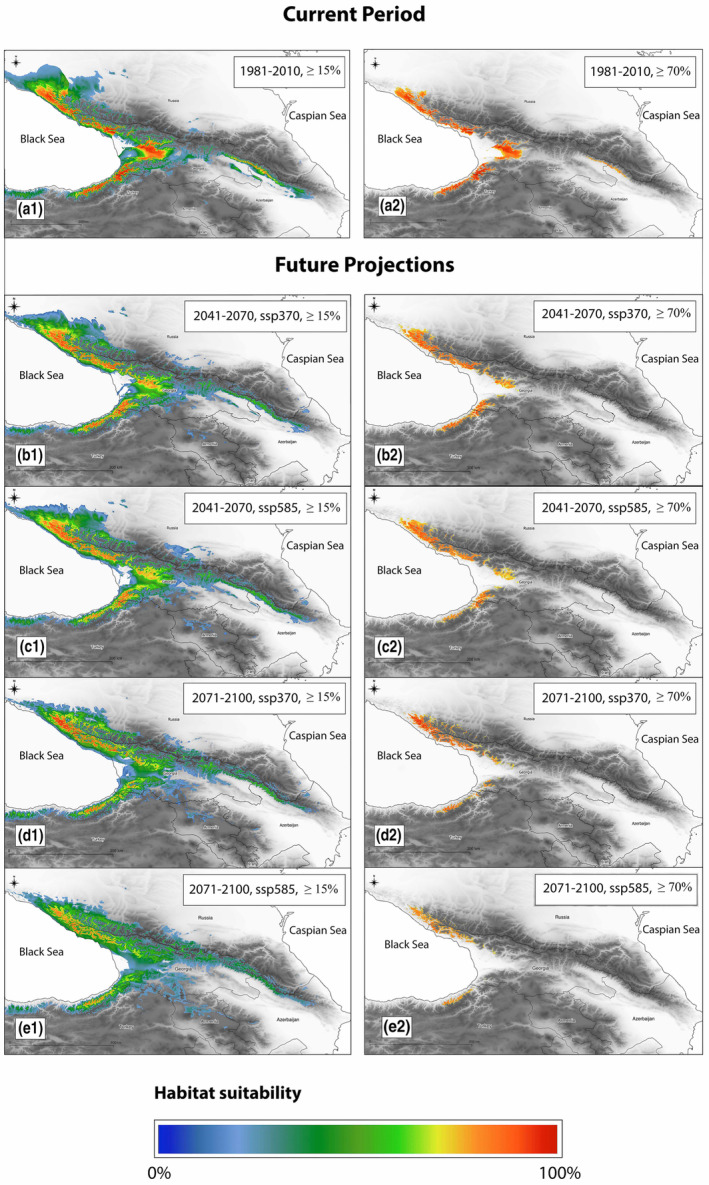
Hypothetical distribution of *Castanea sativa* throughout the region in current (a1‐e1) and future periods (a2‐e2) of time (results averaged across employed models, visit Appendix S2). Two scenarios were applied ‐ ssp370 and ssp585.

The results presented here are averaged for three models used in the analysis; the results for each separate future model (Future 1—GFDL‐ESM4, Future 2—MPI‐ESM1‐2‐HR, and Future 3—UKESM1‐0‐LL) are given in the Appendix S2, Figures S1–S3; Tables S1 and S2. For the current period (1981–2010), the analysis revealed 79,935 km^2^ of total potential distribution with a suitability of at least 15%, mostly distributed in the western part of the studied region (Figure 2a1, Table 4). However, a suitability of 70%, gave only 13,538 km^2^ of range, 16.9% of the total potential area available for the species (Figure 2a2). The highest theoretical altitude for the species to grow reached over 2000 m. a.s.l. (Table 4).

Under scenario ssp370 for 2041–2070, an increase in geographical distribution was predicted (+5667 km^2^) compared with the current period. Areas with high suitability (≥70%) might be lost, especially in the Lesser Caucasus; the exceptions, however, are the Adjara and Pontic Mts. in east Türkiye where the optimal conditions for the sweet chestnut will still be available (Figure 2b2). Also, the eastern part of the range of the sweet chestnut in east Georgia and west Azerbaijan is predicted to disappear. Similarly, the scenario ssp585 for the same period showed an increase in suitable area (12,951 km^2^) for the lower threshold (≥15%) but a decrease in the area with high suitability (Table 4). For 2071–2100, the same scenario predicted an increase in the suitable range (+7541 km^2^) for a threshold ≥15%. However, the area with high suitability would be reduced almost in half, amounting to 7527 km^2^ (Table 4). Scenario ssp585 for the same period showed a slight increase in the total suitable area but predicted a 68% decrease in the highly suitable habitats (Figure 2). Investigating the altitudinal distribution of sweet chestnuts revealed an elevation shift to the higher altitude in response to climate change (Table 4). In the tested scenarios (2041–2070, ssp370 and ssp585; 2071–2100, ssp370 and ssp585) elevation increased to 1596, 1611, 1727, and 1708 m.a.s.l, respectively, compared with ca. 1200 m.a.s.l in the current period.

**FIGURE 3:**
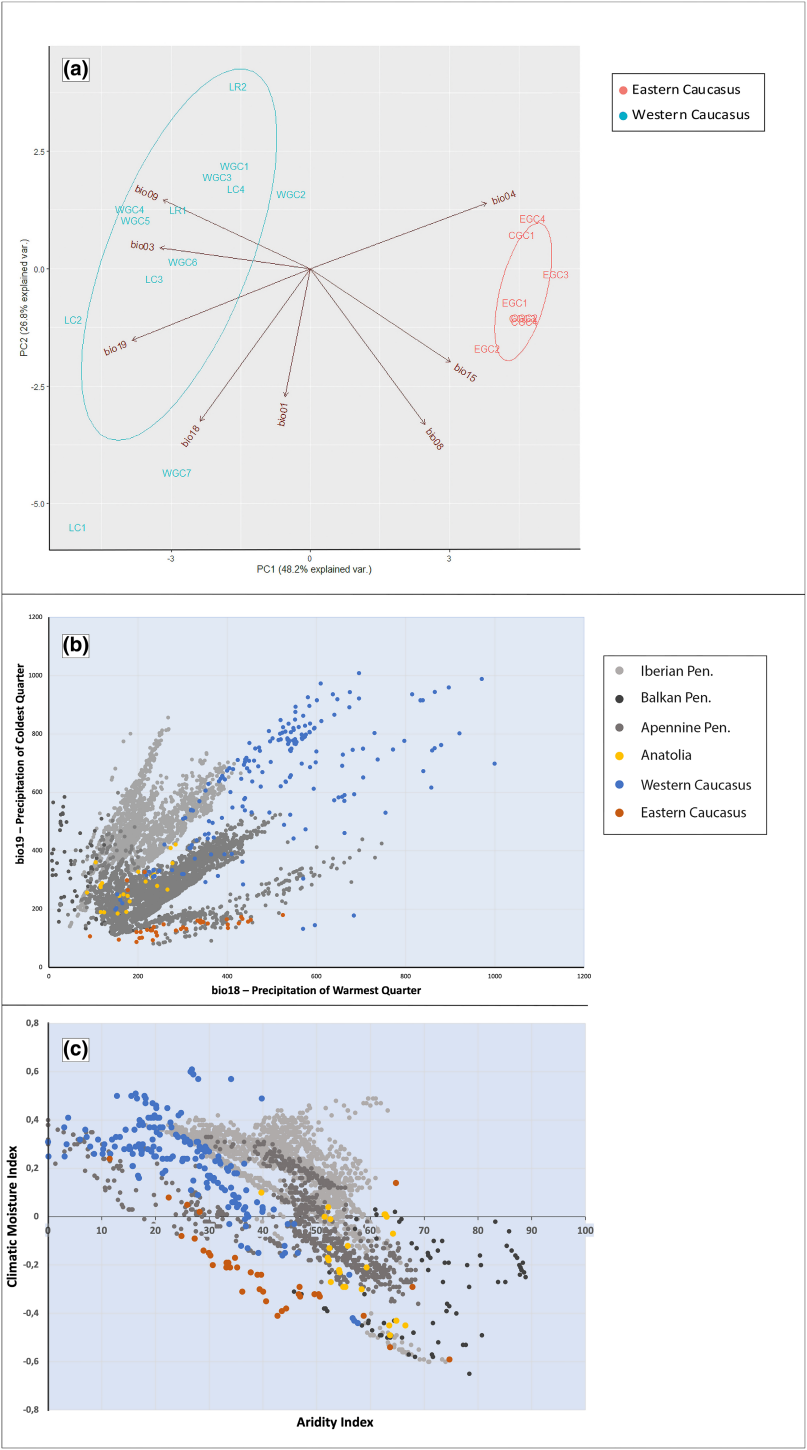
(a) PCA plot built with studied populations of *Castanea sativa* (divided into two groups) and values of bioclimatic variables (see Appendix S2) used in MaxEnt analyses. Ecoplots delivered using (b) bio9 and bio18 variables, and (c) aridity index and climatic moisture index for the whole natural distributional range of species divided into six groups.

Principal component analysis revealed ecological divergence among studied populations and the major driving climatic variables (Figure 3a). The first PC explained 48.2% of the total variation in the data, whereas the second PC explained 26.8%. The variables that loaded positively with PC1 were bio4 (temperature seasonality) and bio15 (precipitation seasonality); in contrast, bio3 (isothermality), bio9 (the mean temperature of the driest quarter) and bio19 (the mean temperature of the driest quarter) loaded negatively with PC1. The variables that loaded strongly negatively with PC2 were bio1 (the annual mean temperature) and bio18 (precipitation of the warmest quarter). Specifically, in the PCA biplot, the first PC separated the Western group (LC, LR, WGC) of sweet chestnut populations from the Eastern group (EGC, CGC) that occupied the opposite area associated with high seasonal precipitation variability and higher thermal seasonality. For the Western subgroup, isothermality (bio3), the mean temperature of the driest quarter (bio9), and precipitation of the coldest quarter (bio19) had the highest positive effect (Figure 3a). PCA indicates that wildly divergent climatic conditions are shared by Western and Eastern populations, mostly related to the contrasting precipitation pattern in both subregions. The exceptionally high values of bio18 and bio19 accounted for the extreme position of two populations LC1 and WGC7. The exact values for bioclimatic variables used are given in Appendix S2, Table S3.

The ecoplot (Figure 3b) visualizes the broad environmental requirements of *C. sativa* in terms of bio18 and bio19 (precipitation in the warmest and coldest quarter, respectively) in the Caucasian and European ranges. Most West Caucasian populations experience much higher precipitation in winter and summer than those in the Iberian and Apennine peninsulas. The Eastern Caucasian populations create a small pocket of climatic space with much lower precipitation values in the warmest and the coldest quarters compared with the Western Caucasian subgroup.

POPS revealed four genetic clusters for the current period (Appendix S1, Figure S4A). West Georgia was shared between two clusters; Cluster 1 (red) grouped populations from the Lesser Caucasus (LC1‐4), and Cluster 2 (blue) stands located in the Likhi Range (LR1 and LR2), Western Greater Caucasus (WGC1‐7) and a single population CGC1 from the Central Greater Caucasus. In the third cluster (brown), populations from the Central Greater Caucasus (CGC2‐4) and Eastern Greater Caucasus (ECG1‐3) were placed, except for population EGC4, which was put in a separate Cluster 4 (green).

**FIGURE 4:**
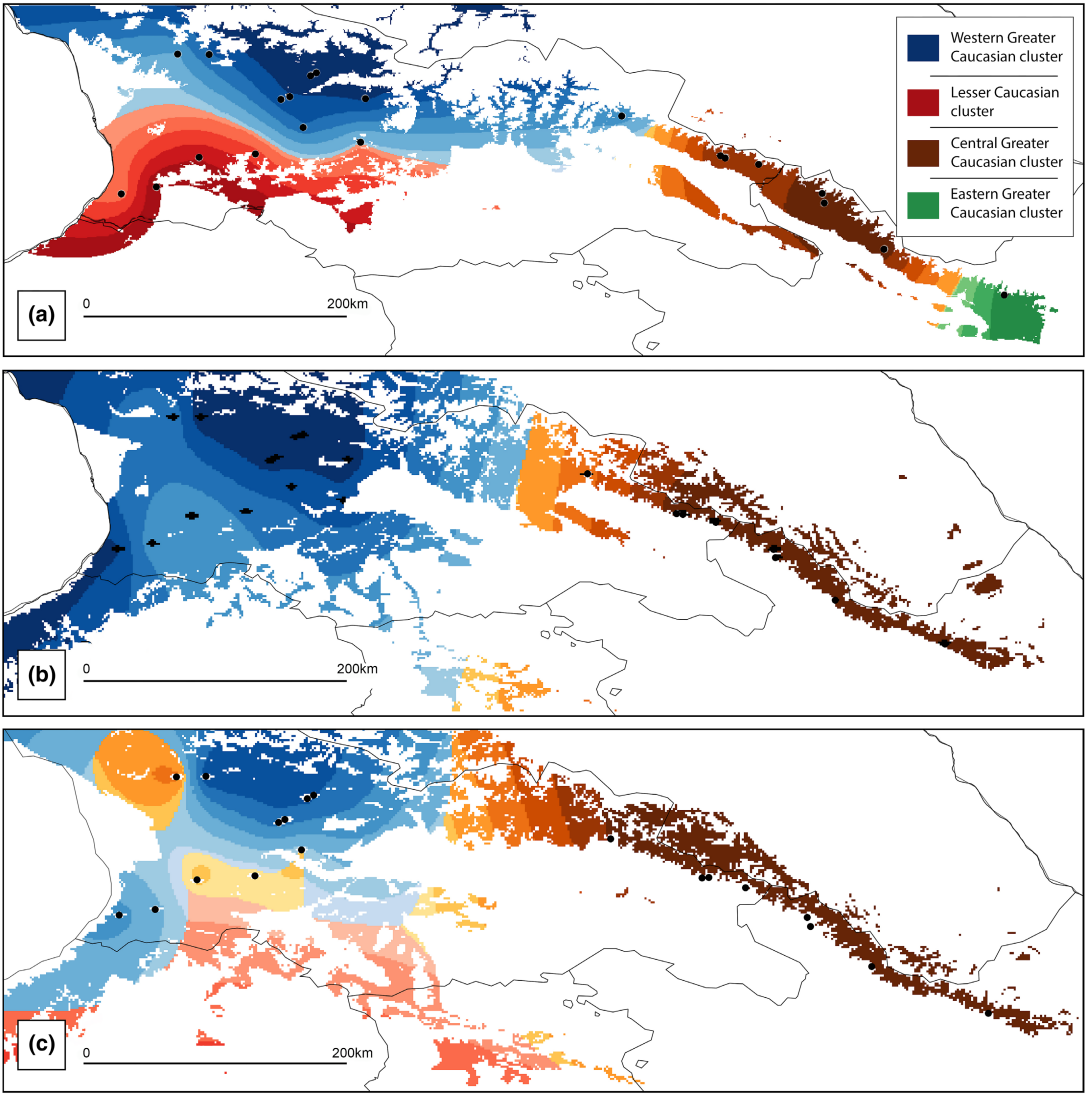
Clustering shifts of *Castanea sativa* neutral genetic structure in response to climate change in the current (1981–2010, a) and the future periods (2071–2100, b—ssp370, c—ssp585). POPS results are overlaid onto MaxEnt hypothetical ranges of the corresponding period and climate change scenario. Black dots denote studied populations.

Spatially explicit simulations showed a loss of variation through time in response to climate changes (Figure 4b,c). The significant future change in the genetic make‐up of populations under ssp370 and ssp585 scenarios denotes the loss of genetic diversity retained in some clusters and their spatial rearrangement that results in higher homogenization of the genetic landscape of the species. Specifically, in the case of ssp370 (Figure 4b), populations from the Lesser Caucasus (Cluster 1) and marginal Eastern Greater Caucasus (Cluster 4) are predicted to disappear from the landscape, whereas the remaining genetic clusters are predicted to expand. However, in the more severe ssp585 climatic scenario (Figure 4c), future population shifts are much more complicated. The erasing of the Eastern Caucasian Cluster 4 is again expected, but this climatic scenario includes the possible expansion of the Central and Eastern Greater Caucasus Cluster III to the Lesser Caucasus and the spatial reduction of Cluster II.

### 3.5 Conservation prioritization

**FIGURE 5:**
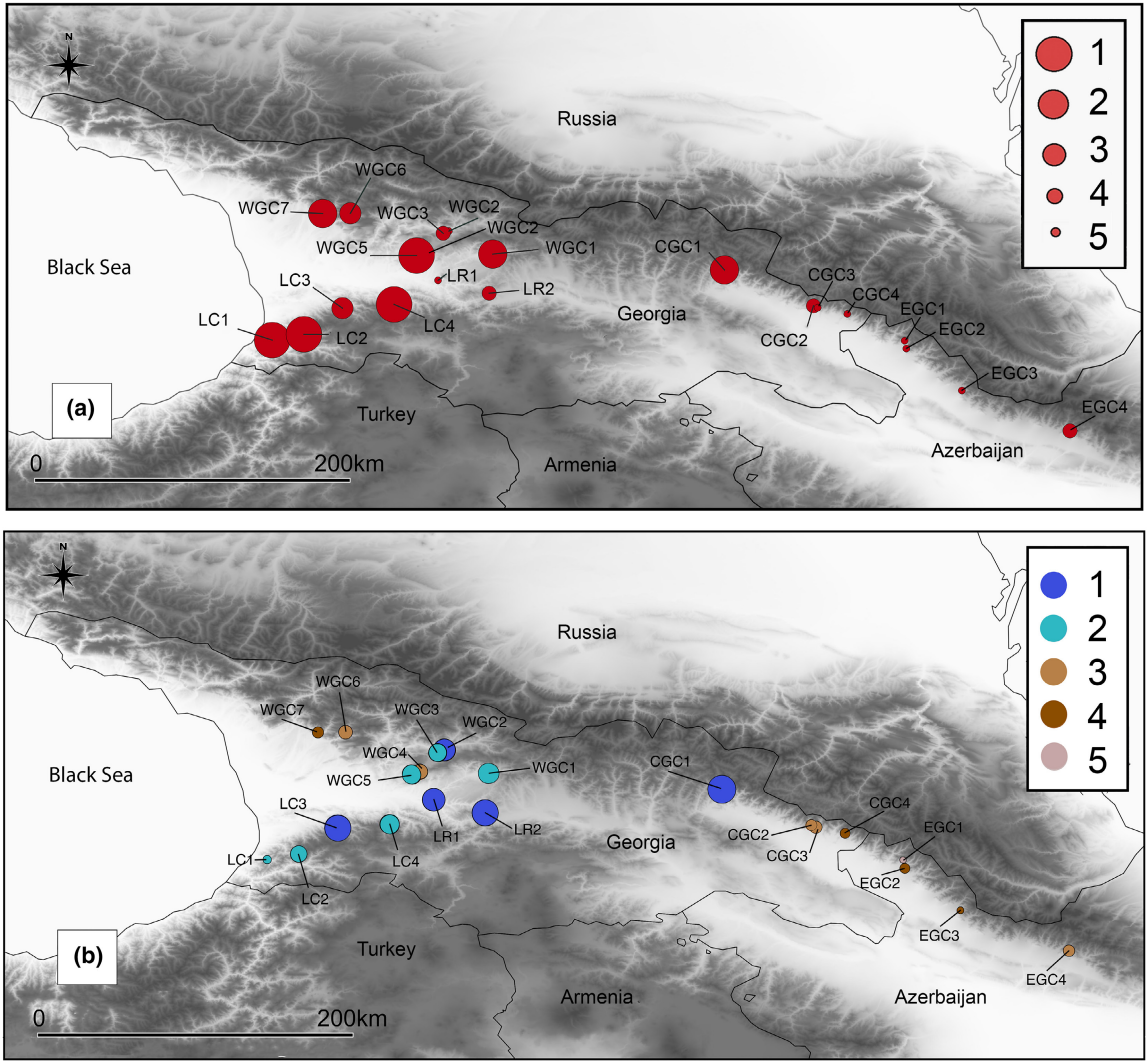
(a) conservation prioritization of *Castanea sativa* natural populations based on allelic distributions (reserve selection analyses; the size of the circle corresponds to the significance for conservation) and (b) conservation prioritization based on *C*_i_ (the significance for conservation is represented with colors and corresponding numbers from 1 to 5).

DIVA‐GIS analysis indicated that populations from the western part of the species range have the highest conservation priority (Figure 5a). Accordingly, the most genetically valuable populations were LC1, LC2, LC4, and WGC5. Other populations with high genetic diversity were WGC7 and WGC1 located in West Georgia, and CGC1 in East Georgia.

A second analysis, which combined genetic and ecological metrics, delivered the same pattern that the western populations are of the highest conservation priority, although it differed in detail. Specifically, populations LC1 and LC2 located in the Lesser Caucasus and WGC5 and WGC7 from the Greater Caucasus lost their high conservation values due to expected significant climate shifts. The exception was population CGC1, which was suggested to experience the lowest climate change among the studied locations. Generally, Central and Eastern Greater Caucasus populations attained a low conservation index. Apart from having decreased genetic diversity, those stands also had low values of ecological indices—relatively low forest continuity and pronounced future climate shift (Appendix S1, Table S1). Overall, the conservation index (Figure 5b; Table 1) ranged from 0.97 (EGC1) to 5.02 (LR2). The highest conservation priority with *C*_i_ category 1 had populations LC3, LR1‐2, WGC2, and CGC1; the next group with *C*_i_ category 2 had LC2, LC4, WGC1, WGC3, and WGC5. The remaining populations had categories from 3 to 5.

## 4 DISCUSSION

### 4.1 Climate as the significant factor driving the genetic structure of *C. sativa* in the South Caucasus

We did not find support for IBC nor IBR for sweet chestnut. The unimportance of ancestry likely reflects the evolutionary history of the species in the region related to Mid‐Pleistocene (Beridze et al., 2023). It is also possible that by using neutral markers, we could not capture the IBC effect in which the adaptive process may be involved (Orsini et al., 2013). In the complex mountain landscape of the Caucasus, we predicted finding at least a weak effect of topography (IBR) since sweet chestnut is insect pollinated (Larue et al., 2021) and the mountainous landscapes would not favor regular connectivity among populations. This has been found in other insect‐pollinated trees such as *Aesculus hippocastanum* L., growing naturally in the Pindos Mts., Greece (Walas et al., 2019). However, it seems that the landscape of the mid‐elevation belt of the Greater and Lesser Caucasus offers functional connectivity among distant patches occupied by populations of sweet chestnut. Also, studies are contradicting previous beliefs that foraging distances of insects are limited (e.g., Kamm et al., 2009; Suchan et al., 2019). Ambophily, that is, coexistence of wind‐ and insect pollination may be an important consideration. Wind pollination can homogenize genetic structure much more effectively than insect pollination since wind may transport sweet chestnut pollen distances over 100 km (Fernández‐López & Alía, 2003). However, the role of long‐distance wind pollination in sweet chestnut is uncertain at the landscape level, as excluding insects can reduce fruit set in plantations by 80% (Larue et al., 2021).

We found that pure climatic conditions (19%) with a confounding effect of geographic distance (21.1%) were responsible for sweet chestnut's extant neutral genetic structure, which denotes the pattern of IBE. Our results also demonstrated that the occurrence of sweet chestnut in the South Caucasus is affected more by precipitation than temperature. The regional climates in the study area, especially precipitation, are controlled by the orography and distance from the Black and Caspian Seas (Nakhutsrishvili & Abdaladze, 2017). The prevailing western winds lose moisture picked up from the Black Sea due to the longitudinal barrier of the Likhi Range. Consequently, *C. sativa* in the western range (West Georgia) and eastern range (East Georgia and Azerbaijan) in the South Caucasus grows under different precipitation conditions. Studies have shown that the neutral genetic diversity of sweet chestnut in the Caucasus and the Iberian Peninsula are organized along the precipitation gradient (Beridze et al., 2023; Míguez‐Soto et al., 2019). Beridze et al. (2023) demonstrated a reduced gene flow between two major clusters covering the western and eastern parts of the species range in the Caucasus, corresponding to the two climatic groups defined in this study. The selection against immigrants sourced in divergent ecological gradients or/and reduced fitness of hybrids may lead to IBE (Wang & Bradburd, 2014). Both phenomena may also contribute to limited genetic connectivity between western and eastern clusters, driving the current genetic structure of *C. sativa* in the region.

### 4.2 Future occurrence and distribution of neutral genetic structure

At first glance, the hypothetical range of *C. sativa* in the Caucasus is expanding in future. However, a closer look reveals a reduction in highly preferable areas (≥70% suitability) in 2041–2070. Next, up to 2100, we predict even worse effects of climate change for the extreme ssp585 scenario with an almost 70% loss. Range contraction would likely be most drastic in Eastern Georgia and Azerbaijan, putting the population at the easternmost locations at risk of extinction. Similar to other global hotspots of diversity, the Caucasus is considered highly vulnerable to the adverse effects of climate change (IPCC, 2021). Niche modeling studies repeatedly show a rising risk of habitat loss for Caucasian plants (Dering et al., 2021; Sękiewicz et al., 2022; Zazanashvili et al., 2011) and animals (Gül et al., 2018; Hof & Allen, 2019) as a result of the ongoing climate crisis. In the last two decades, a significant shift toward higher temperatures and lower precipitation has been observed in Georgia and Azerbaijan (CCKP, 2022; Elizbarashvili et al., 2017). The current direction of climate change may likely amplify the already existing threats and make sweet chestnut more prone to biotic and abiotic hazards.

The results also suggest that the climatically suitable habitat for *C. sativa* will be displaced to higher altitudes, as generally predicted (IPCC, 2007; Vitasse et al., 2021). Currently, sweet chestnut reaches a maximal occurrence at 1450 m a.s.l in Georgia and 1700 m a.s.l. in Azerbaijan. The expected maximal upwards redistribution of the suitable habitats for sweet chestnut based on our SDMs estimations is predicted to be ca. 350 m in 60 years or ca. 50 m per decade. In comparison, the reported average global upwards shift for plant species is between 3 and 11 m per decade (Chen et al., 2011; Pauli et al., 2012; Savage & Vellend, 2015), far below that predicted for sweet chestnut. The combined interaction of various biotic and abiotic factors controls the range expansion (Corlett & Westcott, 2013; Gougherty et al., 2021; Pauli et al., 2012). Consequently, the theoretical habitats projected at higher altitudes may not be accessible for sweet chestnut to trace climate changes leading to even greater range's shrinkage.

Our modeling of genetic structure changes may serve as a rough approximation of the changes in adaptive genetic diversity because the relationships between neutral and adaptive genetic diversity are not straightforward (Dauphin et al., 2020). According to our results, the forecasted climate changes may markedly redistribute the current neutral genetic structure of the species across the landscape. We predict a loss of two genetic clusters, which undoubtedly may reduce the variability of the sweet chestnut gene pool and deprive it of beneficial alleles. The homogenization of the western portion of the sweet chestnut range, where the species is most abundant, and populations contain a high number of private alleles and the highest levels of gene diversity (Beridze et al., 2023), is of significant concern. The broadest spectrum of genetic diversity is essential for species to maintain demographic stability while coping with environmental changes. Additionally, POPS suggested the extinction of the easternmost genetic cluster, representing sweet chestnut's peripheral and climatically marginal location in the Caucasus. Considering that the eastern populations grow in distinct climatic conditions in comparison to western populations, we may expect local adaptation there, which may be at serious risk of extinction under the climate change trajectory projected in our study.

### 4.3 Conservation prioritization

As recently demonstrated by Schmidt et al. (2022), the extinction risk assessment available on the IUCN Red List weakly supports the decision‐making of conservation efforts. Accordingly, sweet chestnut is described as “Least Concern,” which reflects its conservation status in Europe. The Caucasian range is an isolated and remote enclave, disjunct from the main European range that practically precludes the beneficial effects of gene flow. Unless special conservation management is considered, such as assisted gene flow or migration, the species' resilience and persistence in the Caucasus in the face of the current climate crisis rely on the standing genetic diversity of local populations.

The high values of historical *N*_eCOAL_ based on the coalescence approach, suggest that demographic stochasticity was not a dominating factor in the long‐term evolutionary history of the studied populations. This agrees with other investigations demonstrating the wide distribution of sweet chestnut in the past in the South Caucasus (Shatilova et al., 2011). On the contrary, the assessed *N*_eLD_ values, which refer to the last few generations and inform about near‐term genetic erosion risk, are much below the recommended value of 500 (Hoban et al., 2020), needed to secure population resilience and adaptation. Accordingly, all studied populations, except for WGC3, are defined as being at risk of loss of adaptive potential because of critically low *N*_e_. However, we know that accurate estimates of *N*_e_ may be especially difficult in trees because some aspects of their population biology violate methodological assumptions of *N*_e_ estimation (Santos‐del‐Blanco et al., 2022). The values presented here may be down‐biased because of spatially restricted sampling and the intensive gene flow (Beridze et al., 2023). Also, trees are assumed to be able to efficiently respond to selection despite low *N*_e_ (Hoban et al., 2021) because of a unique set of life history traits and genetic architecture allowing them for rapid adaptation (Aitken et al., 2008; Petit & Hampe, 2006).

By integrating genetic and ecological metrics, we could effectively prioritize sweet chestnut populations suitable for ex situ and in situ conservation. Such a combined approach is necessary to capture and protect the broadest spectrum of species diversity in future. Accordingly, we observed the highest accumulation of allelic diversity in West Georgia, especially in populations from the West Lesser Caucasus, and the lowest toward Azerbaijan. Arguably, this pattern is partly an imprint of the species' glacial history—the proximity to the LGM refugial areas in Colchis (Beridze et al., 2023; Mahler et al., 2022). A similar pattern was reported for other trees in the region, which were excluded from direct human impact (Christe et al., 2014; Maharramova et al., 2018; Sękiewicz et al., 2022). We presume that more optimal conditions prevailing in the western portion of the range buffered the populations against the genetic drift. Consequently, westerly located populations, particularly LC1, LC2, LC4, and WGC5, were captured by the Reserve Selection procedure with the highest priority for conservation. The lowest indices of *A*_r_ and *A*_p_ were noted in the eastern populations. Given the low human impact on sweet chestnut in the South Caucasus, we assume that low genetic diversity is attributed to natural processes induced by a more suboptimal climate. More demanding climate conditions in the eastern range (lower precipitation and higher temperatures) could have intensified drift and selection; if strong enough, selection may also lead to neutral diversity loss (Jin et al., 2022).

The complementary approach using the modified *C*_i_ redefines the priority status of the studied populations. The two populations from the Likhi Range (LR1 and LR2) had the highest category of *C*_i_. These have a relatively high genetic diversity and are within continuous forest, but the current climate conditions also overlap with the future ones, giving a high probability of in situ survival. Priority 2 of *C*_i_ was ascribed to population CGC1 from the Greater Caucasus, which in the Reverse Selection was also considered for conservation due to high allelic diversity; here, the environmental indices and climate predictions were relatively high, giving justified prospects for future survival. Sweet chestnut populations from the Lesser Caucasus in West Georgia with the highest allelic diversity acquired low *C*_i_. At those locations, the climate components are expected to shift too drastically in future, which questions their in situ survival. For LC1 and LC2, precipitation values of the warmest quarter are projected to decrease by ca. 52% and 70%, respectively, and precipitation of the coldest quarter by ca. 28% and 56%, respectively. POPs also predicted a loss of the genetic cluster containing populations from this part of the species range. However, the highest allelic diversity noted in LC1 and LC2 makes them perfect objects for ex situ conservation or using advanced methods such as assisted migration. The populations from the eastern locations, except for CGC1, were classified as of marginal attention in conservation management. Besides possessing low genetic diversity indices, the future climate will likely fail to provide suitable habitats, precluding their in situ persistence. Therefore, the easterly located populations may face the greatest vulnerability to climate change and the most significant risk of future extirpation. Ex situ conservation actions may be the only way to preserve those genetic resources.

### 4.4 Final remarks

Only a tiny part of the sweet chestnut populations studied here is within the networks of protected areas in Georgia and Azerbaijan (Figure 1). *Castanea sativa* is already declining in the South Caucasus (Dumbadze et al., 2018; Tavadze et al., 2013) acknowledged by putting it on the red lists of both countries. In the most pessimistic scenario, we predicted a 70% reduction in the overall distribution, including a one‐third reduction in the highly suitable areas for the species. These estimations refer only to climate‐led reductions. At the same time, the region struggles with the high pressure of agriculture and urbanization and the lack of legal regulations and funds for nature conservation. Forests are under pressure from illegal and unsustainable logging, fragmentation, and poor management (NBSAP‐2, MoEPA, 2014). Despite the growing awareness of climate change on natural and human systems (e.g., Durglishvili & Kechakmadze, 2020), the necessity of protecting biodiversity is poorly appreciated in the region (6NR‐Georgia, 2020).

The relatively wide niche breadth of *C. sativa* in the South Caucasus suggests that at least some populations are already preadapted for novel climates. Quantifying the amount of preadaptation or maladaptation to the expected novel climates relies on an adaptive genomic approach, which maps the populations with the highest potential performance in new conditions. This approach has already been applied to other tree species (e.g., Gougherty et al., 2021). It has great potential in nature conservation to advance beyond static concepts of diversity protection toward tailored and precise actions aiming for efficient management in the reality of the ongoing climate crisis, especially in such exceptional regions like hotspots of diversity.

## AUTHOR CONTRIBUTIONS

**Berika Beridze:** Conceptualization (supporting); data curation (equal); formal analysis (equal); investigation (equal); methodology (equal); software (equal); visualization (equal); writing – original draft (equal); writing – review and editing (equal). **Katarzyna Sękiewicz:** Conceptualization (equal); data curation (equal); formal analysis (equal); funding acquisition (supporting); investigation (equal); methodology (equal); project administration (supporting); resources (supporting); software (equal); supervision (supporting); validation (equal); visualization (equal); writing – original draft (supporting); writing – review and editing (equal). **Łukasz Walas:** Data curation (supporting); formal analysis (equal); methodology (supporting); resources (supporting); software (supporting); supervision (supporting); validation (supporting); visualization (supporting); writing – original draft (supporting); writing – review and editing (supporting). **Peter A. Thomas:** Conceptualization (supporting); investigation (equal); resources (equal); validation (equal); writing – review and editing (equal). **Irina Danelia:** Funding acquisition (supporting); methodology (supporting); resources (equal); validation (equal); writing – review and editing (supporting). **Vahid Fazaliyev:** Funding acquisition (supporting); methodology (supporting); resources (equal); writing – review and editing (supporting). **Giorgi Kvartskhava:** Funding acquisition (supporting); methodology (supporting); writing – review and editing (supporting). **Jan Sós:** Writing – review and editing (supporting). **Monika Dering:** Conceptualization (lead); data curation (equal); formal analysis (supporting); funding acquisition (lead); investigation (equal); methodology (equal); project administration (lead); resources (lead); software (equal); supervision (lead); validation (equal); visualization (equal); writing – original draft (equal); writing – review and editing (equal).

## Supporting information


Appendix S1

Appendix S2

## Data Availability

The data that supports the findings of this study are available in the supplementary material of this article. Data is available on request from the authors.
