# Supplementary material for: Biodiversity protection against anthropogenic climate change: Conservation prioritization of Castanea sativa in the South Caucasus based on genetic and ecological metrics
Source: Ecol Evol. 2023 May 18;13(5):e10068. doi: 10.1002/ece3.10068 (PMC10196223; doi:10.1002/ece3.10068)
Supplement: Supplementary file 1 — Appendix S1 [file ECE3-13-e10068-s002.docx]

**Appendix S1: Supplementary file LAN**

**Biodiversity protection against anthropogenic climate change: conservation prioritisation of  *Castanea sativa*in the South Caucasus based on genetic and ecological metrics**

Berika Beridze^1,2^, Katarzyna Sękiewicz^1^, Łukasz Walas^1^, Peter A. Thomas^3^, Irina Danelia^4,5^, Vahid Fazaliyev^6^, Giorgi Kvartskhava^5^, Jan Sós^7^, Monika Dering^1,7*^

1. Institute of Dendrology, Polish Academy of Sciences, Parkowa 5, 62-035, Kórnik, Poland

2. Adam Mickiewicz University in Poznań, Faculty of Biology, Wieniawskiego 1, Poznań, Poland

3. School of Biological Sciences, Keele University, Staffordshire, ST5 5BG, United Kingdom

4. National Botanical Garden of Georgia, Botanikuri Street 1, Tbilisi, Georgia

5. Faculty of Agricultural Science and Bio-System Engineering, Georgian Technical University, Guramishvili Str. 17, Tbilisi, Georgia

6. Forest Development Service, Ministry of Ecology and Natural Resources of Azerbaijan, Baku, Azerbaijan

7. Poznań University of Life Sciences, Department of Silviculture, Wojska Polskiego 71c, 61-625, Poznań, Poland

* Corresponding author: monika.dering@up.poznan.pl


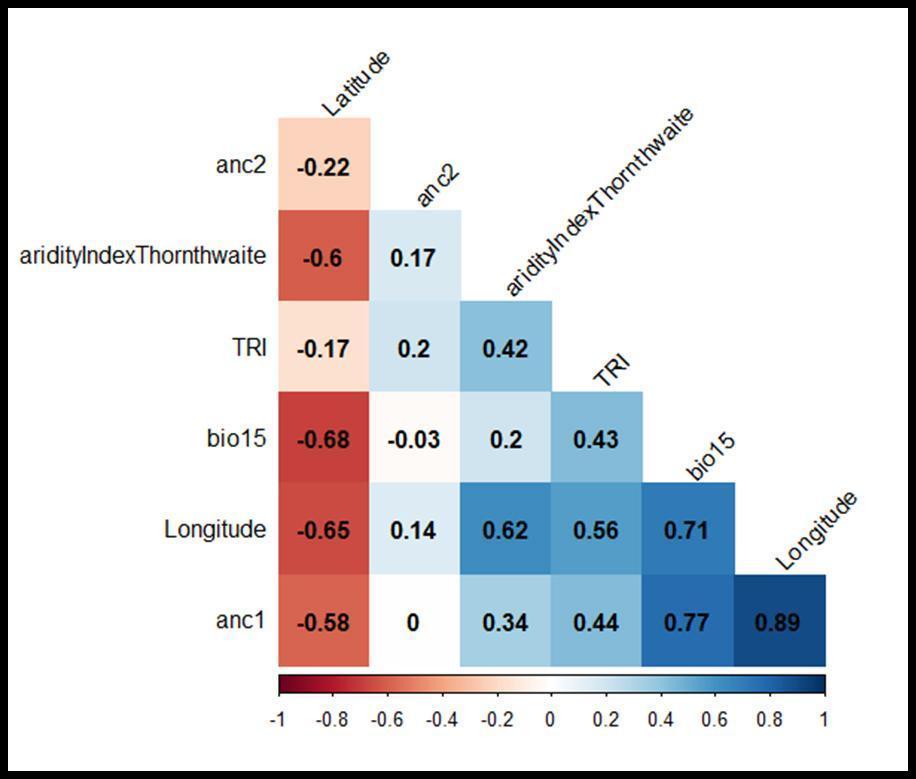

Figure S1. Correlation between explanatory variables employed in dbRDA analyses; anc1 and anc2 - first and second principal component from PCA conducted on Q-values (cluster membership) obtained from STRUCTURE results (Beridze et al., 2023); TRI - terrain ruggedness index, bio15 - precipitation seasonality, aridity index.


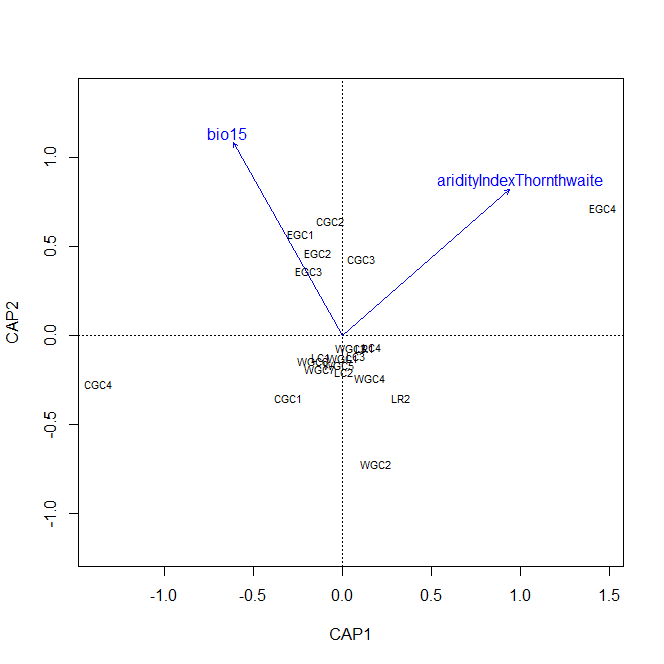


Figure S2. PCA plot of studied populations and two significant environmental variables associated with genetic variation (bio15 - precipitation seasonality and aridity index).

**
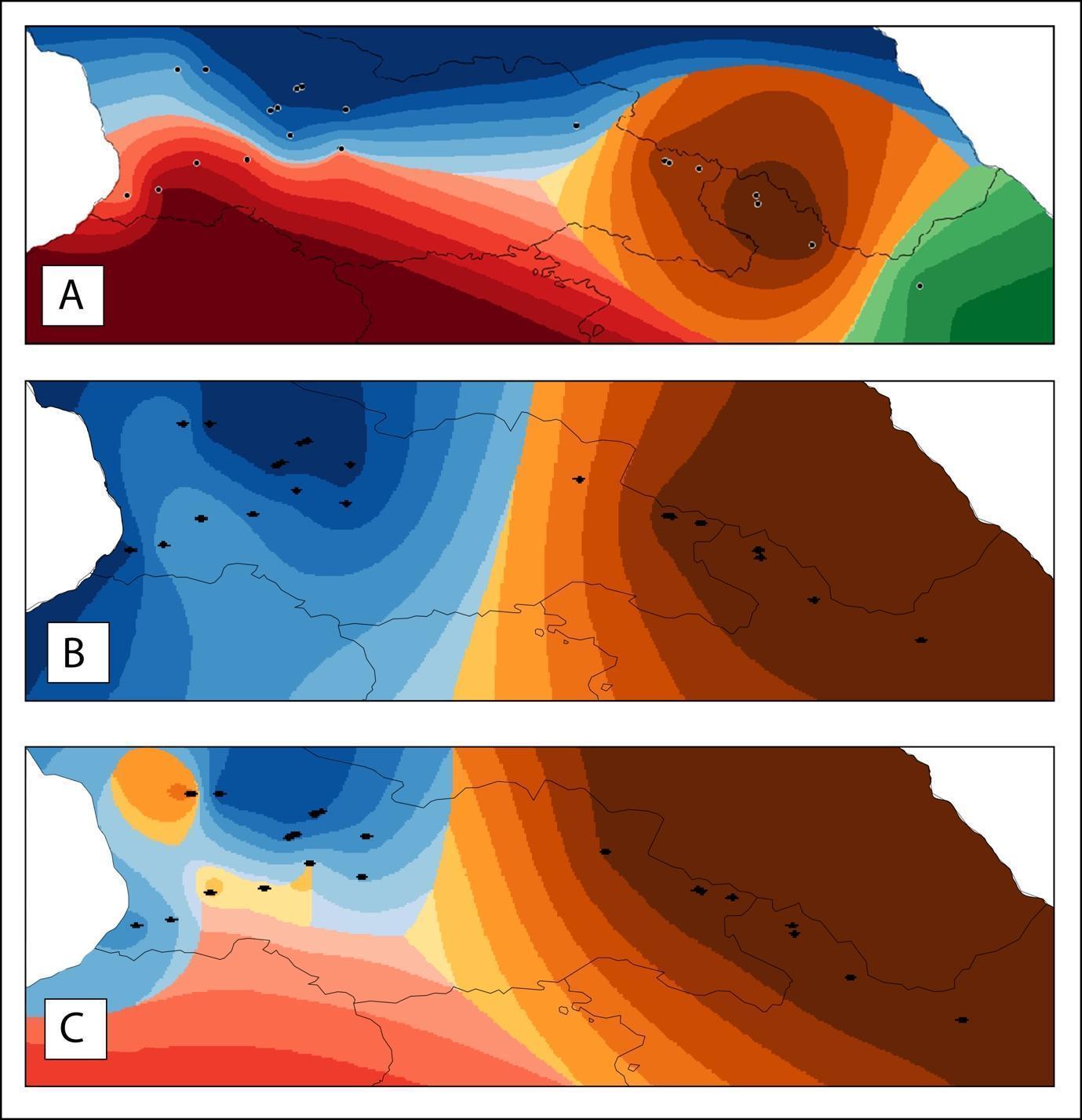
**

Figure S3. The raw output files from POPS depicting the current population clustering (A, 1981-2010) and future population membership shift in response to climate change (B – sspp370, C – ssp585; 2071-2100) throughout the whole landscape. Black dots represent location of sampled sweet chestnut populations. Blue colour – Western Greater Caucasus cluster; Red – Lesser Caucasus; Brown – and Green –  Central Greater and Eastern Greater Caucasus clusters, respectively.


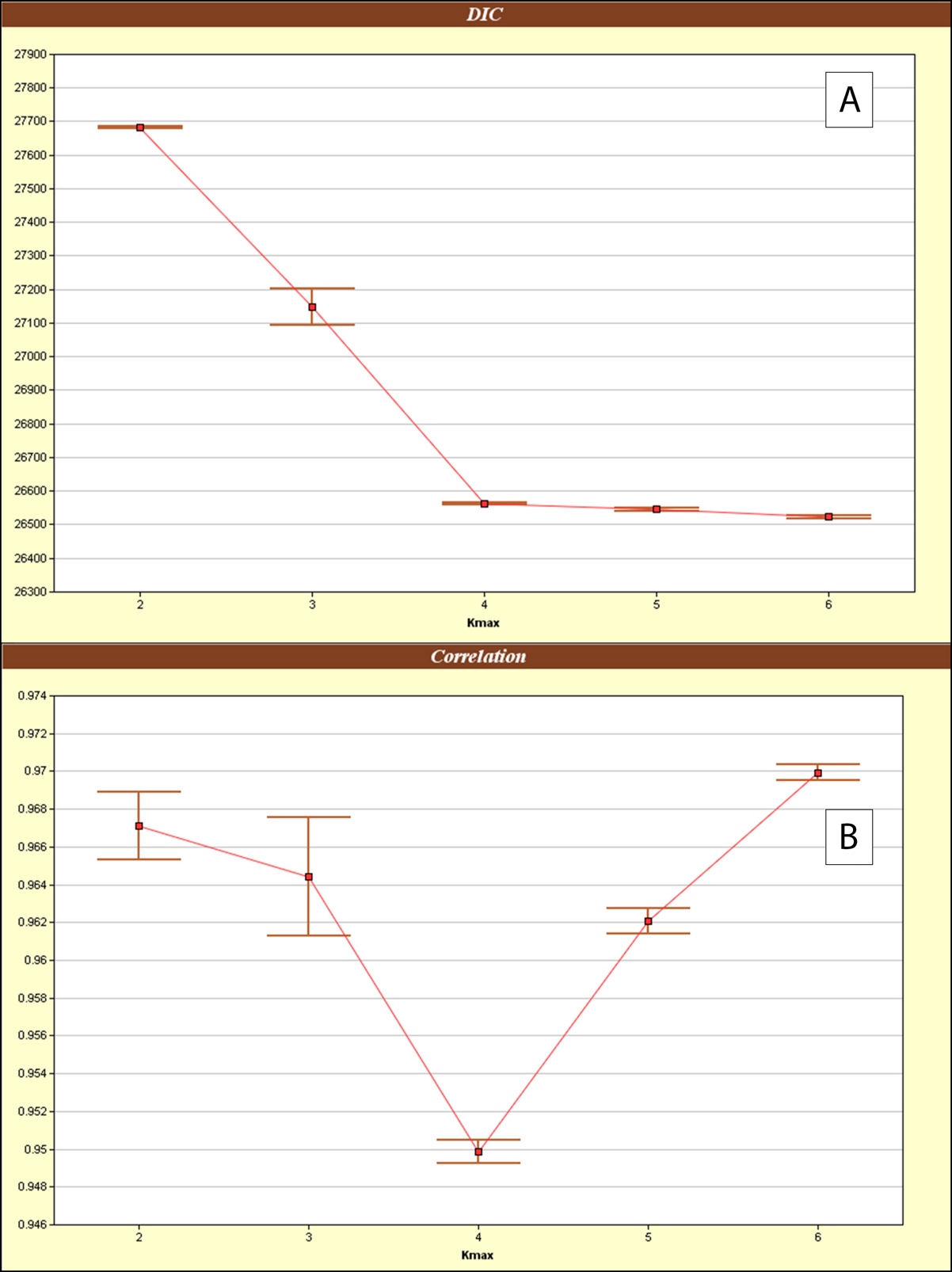

Figure S4. The best K and the best run of POPS analyses were chosen according to (A) DIC and (B) correlation values.

Table S1. Conservation index delivered for 21 natural populations of *Castanea sativa*.

| Pop | *Ar* | *Fc* | *CI* | *Ci* |
| --- | --- | --- | --- | --- |
| LR2 | 4.42 | 73.57 | 64.74 | 5.02 |
| CGC1 | 4.64 | 51.81 | 51.59 | 4.66 |
| LC3 | 5.60 | 69.63 | 84.54 | 4.61 |
| WGC2 | 4.58 | 75.17 | 79.67 | 4.32 |
| LR1 | 5.60 | 70.20 | 95.65 | 4.11 |
| WGC1 | 4.62 | 63.16 | 76.16 | 3.83 |
| WGC3 | 4.80 | 68.76 | 87.46 | 3.77 |
| WGC5 | 5.64 | 81.10 | 121.60 | 3.76 |
| LC4 | 5.62 | 65.05 | 100.87 | 3.62 |
| LC2 | 5.51 | 89.15 | 158.05 | 3.11 |
| WGC4 | 4.20 | 83.88 | 124.86 | 2.82 |
| CGC3 | 4.38 | 38.44 | 67.97 | 2.48 |
| WGC6 | 4.92 | 80.35 | 164.67 | 2.40 |
| CGC2 | 3.88 | 39.73 | 69.79 | 2.21 |
| EGC4 | 3.24 | 32.78 | 52.80 | 2.01 |
| WGC7 | 5.39 | 73.55 | 198.64 | 1.99 |
| CGC4 | 3.65 | 40.88 | 84.69 | 1.76 |
| EGC2 | 3.66 | 49.11 | 108.67 | 1.65 |
| LC1 | 5.86 | 60.98 | 268.90 | 1.33 |
| EGC3 | 3.20 | 27.62 | 74.93 | 1.18 |
| EGC1 | 3.85 | 27.50 | 109.54 | 0.97 |

Pop – population identity, *Ar* – allelic richness, *Fc* – forest cover in percentages, *Cl* – geometric mean between current and future climatic conditions (bioclimatic variables), *Ci* – conservation index.
