# Supplementary material for: Biodiversity protection against anthropogenic climate change: Conservation prioritization of Castanea sativa in the South Caucasus based on genetic and ecological metrics
Source: Ecol Evol. 2023 May 18;13(5):e10068. doi: 10.1002/ece3.10068 (PMC10196223; doi:10.1002/ece3.10068)
Supplement: Supplementary file 2 — Appendix S2 [file ECE3-13-e10068-s001.docx]

**Appendix S2: Supplementary file SDM**

**Biodiversity protection against anthropogenic climate change: conservation prioritisation of  *Castanea sativa*in the South Caucasus based on genetic and ecological metrics**

Berika Beridze^1,2^, Katarzyna Sękiewicz^1^, Łukasz Walas^1^, Peter A. Thomas^3^, Irina Danelia^4,5^, Vahid Fazaliyev^6^, Giorgi Kvartskhava^5^, Jan Sós^7^, Monika Dering^1,7*^

1. Institute of Dendrology, Polish Academy of Sciences, Parkowa 5, 62-035, Kórnik, Poland

2. Adam Mickiewicz University in Poznań, Faculty of Biology, Wieniawskiego 1, Poznań, Poland

3. School of Biological Sciences, Keele University, Staffordshire, ST5 5BG, United Kingdom

4. National Botanical Garden of Georgia, Botanikuri Street 1 Street, Tbilisi, Georgia

5. Faculty of Agricultural Science and Bio-System Engineering, Georgian Technical University, Guramishvili Str. 17, Tbilisi, Georgia

6. Forest Development Service, Ministry of Ecology and Natural Resources of Azerbaijan, Baku, Azerbaijan

7. Poznań University of Life Sciences, Department of Silviculture, Wojska Polskiego 71c, 61-625, Poznań, Poland

* Corresponding author: monika.dering@up.poznan.pl

Figure S1. Future habitat suitability area of *C. sativa* in the Caucasus ecoregion predicted by MAXENT software using GFDL-ESM4 CMIP6 model.
A1_ 2041-2070, ssp370, ≥ 15% threshold, A2 _ 2041-2070, ssp370, ≥ 70% threshold;
B1_ 2041-2070, ssp585, ≥ 15% threshold, B2 _ 2041-2070, ssp585, ≥ 70% threshold;
C1_ 2071-2100, ssp370, ≥ 15% threshold, C2 _ 2071-2100, ssp370, ≥ 70% threshold;
D1_ 2071-2100, ssp585, ≥ 15% threshold, D2 _ 2071-2100, ssp585, ≥ 70% threshold.


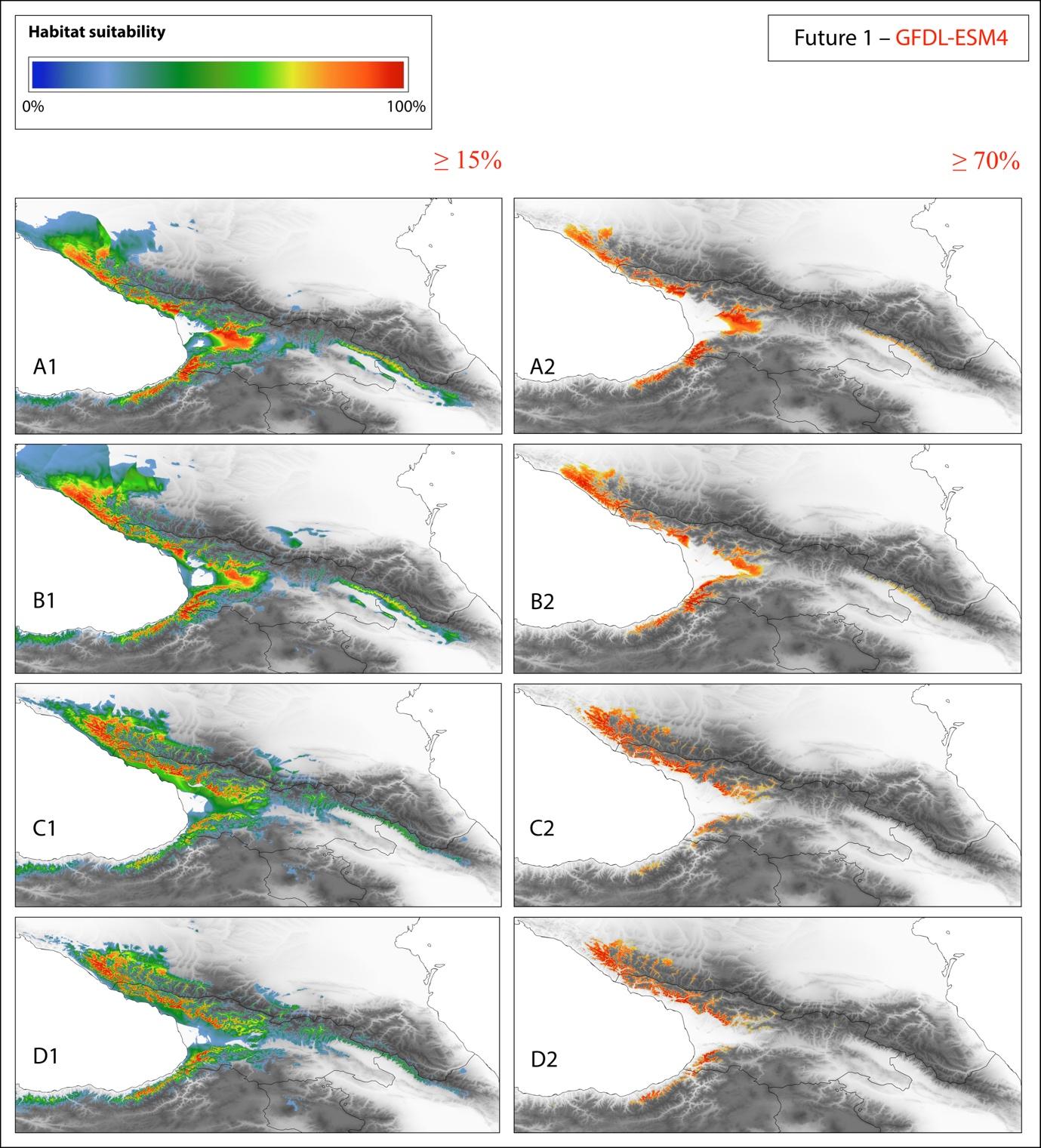


Figure S2. Future habitat suitability area of *C.* sativa in the Caucasus ecoregion predicted by MAXENT software using MPI-ESM1-2HR CMIP6 model.
A1_ 2041-2070, ssp370, ≥ 15% threshold, A2 _ 2041-2070, ssp370, ≥ 70% threshold;
B1_ 2041-2070, ssp585, ≥ 15% threshold, B2 _ 2041-2070, ssp585, ≥ 70% threshold;
C1_ 2071-2100, ssp370, ≥ 15% threshold, C2 _ 2071-2100, ssp370, ≥ 70% threshold;
D1_ 2071-2100, ssp585, ≥ 15% threshold, D2 _ 2071-2100, ssp585, ≥ 70% threshold.


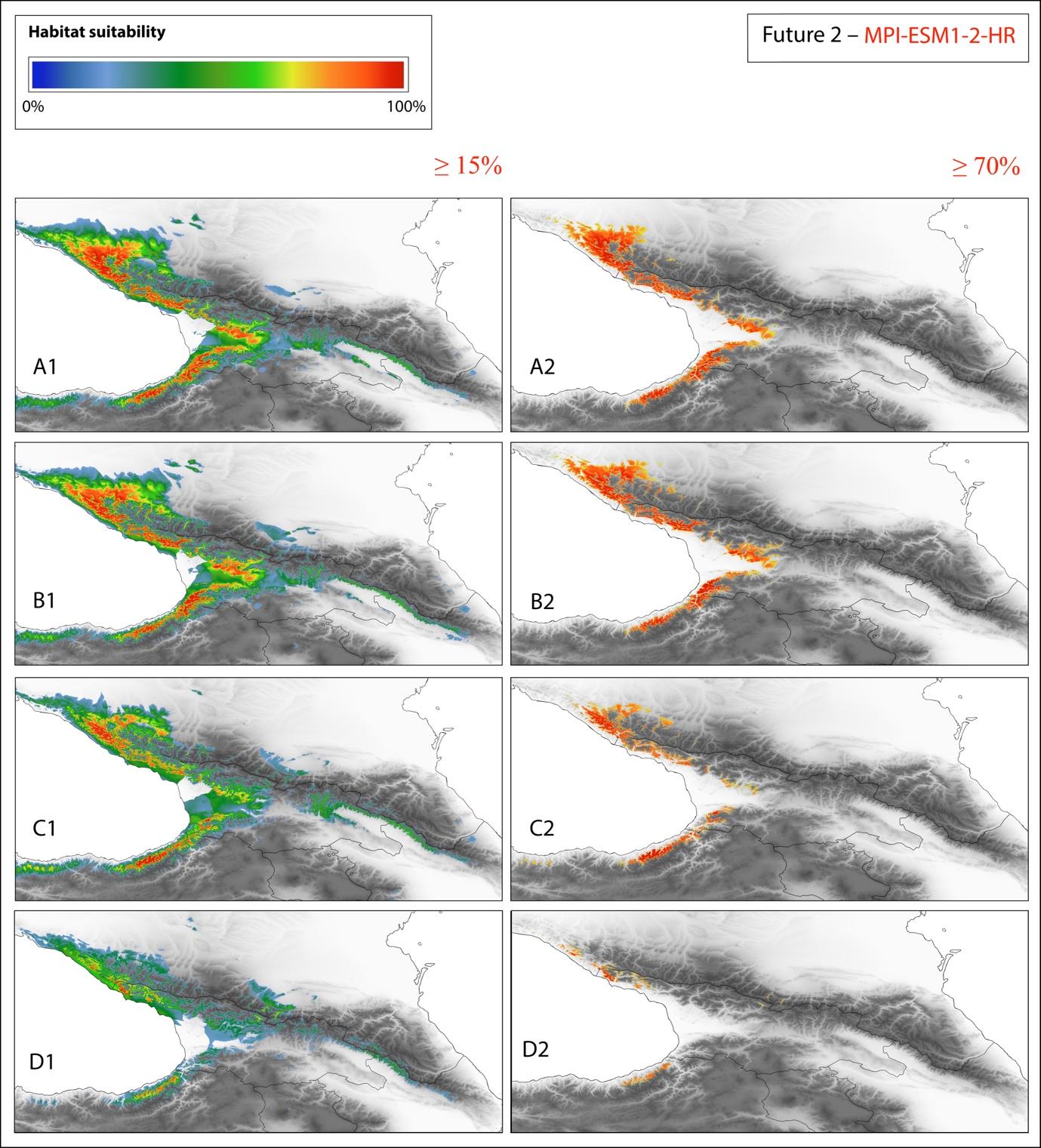


Figure S3. Future habitat suitability area of *C. sativa* in the Caucasus ecoregion predicted by MAXENT software using only UKESM1-0-LL CMIP6 model.
A1_ 2041-2070, ssp370, ≥ 15% threshold, A2 _ 2041-2070, ssp370, ≥ 70% threshold;
B1_ 2041-2070, ssp585, ≥ 15% threshold, B2 _ 2041-2070, ssp585, ≥ 70% threshold;
C1_ 2071-2100, ssp370, ≥ 15% threshold, C2 _ 2071-2100, ssp370, ≥ 70% threshold;
D1_ 2071-2100, ssp585, ≥ 15% threshold, D2 _ 2071-2100, ssp585, ≥ 70% threshold.


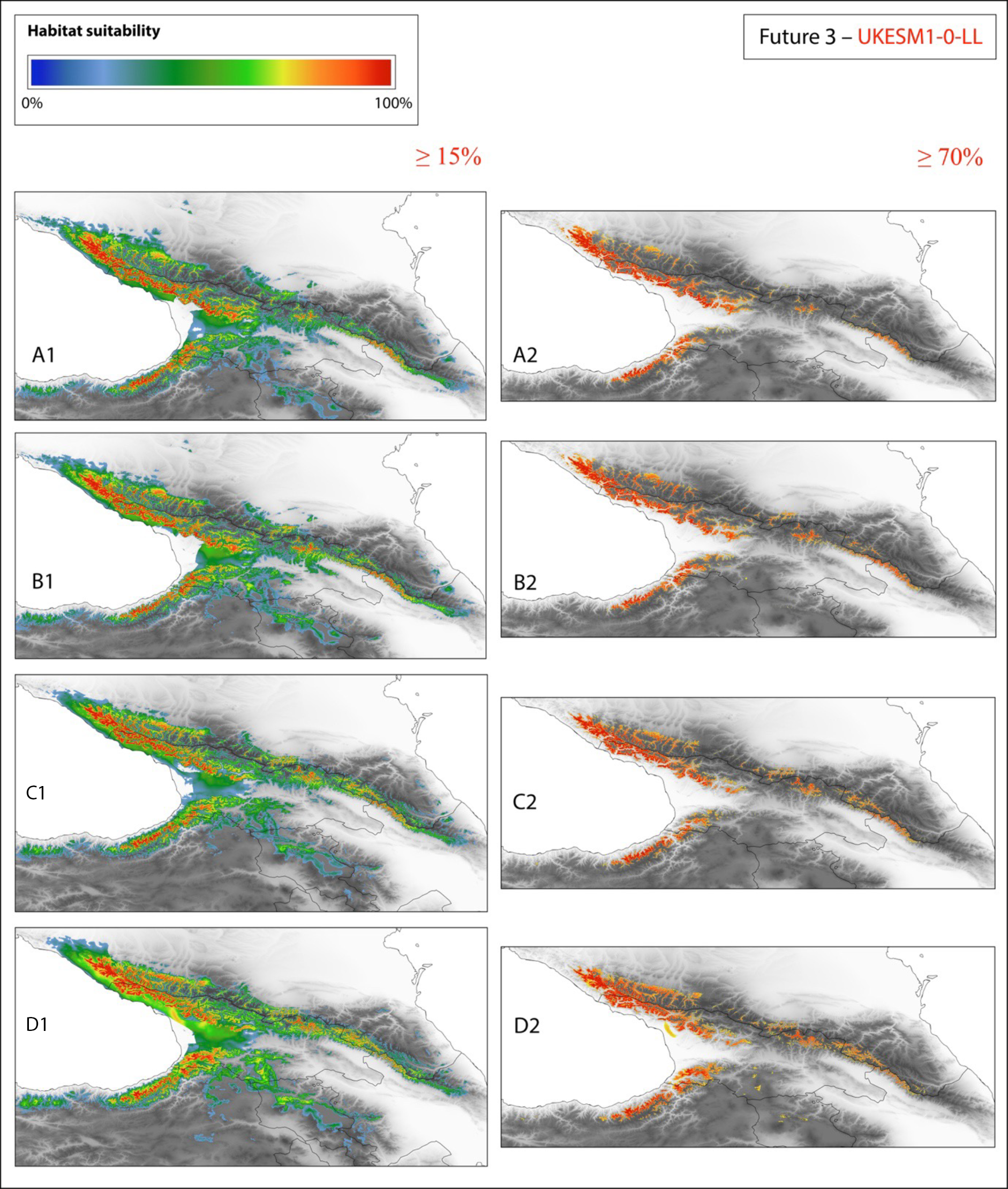


Table S1. The area under the curve (AUC) values, future distributional area for future periods based on each models used.

|  | Time interval | Change scenario | *AUC* | **Total area of distribution km²** | Change in area distribution** km² | **Distribution area ≥ 70% km²** | Change in area distribution ( ≥ 70%) km² |
| --- | --- | --- | --- | --- | --- | --- | --- |
| **Future 1** | 2041 - 2070 | ssp370 | 0.972 | 75441 | -4494 | 13241 | -297 |
|  |  | ssp585 | 0.970 | 89358 | +9423 | 13716 | +178 |
| GFDL - ESM4 | 2071 - 2100 | ssp370 | 0.971 | 74290 | -5645 | 10632 | -2906 |
|  |  | ssp585 | 0.970 | 18604 | -61331 | 9631 | -3907 |
| **Future 2** | 2041 - 2070 | ssp370 | 0.972 | 77005 | -2930 | 15727 | +2189 |
|  |  | ssp585 | 0.970 | 80864 | +929 | 15784 | +2246 |
| MPI - ESM1 - 2 - HR | 2071 - 2100 | ssp370 | 0.972 | 69560 | -10375 | 8720 | -4818 |
|  |  | ssp585 | 0.972 | 54010 | -25925 | 1963 | -11575 |
| **Future 3** | 2041 - 2070 | ssp370 | 0.973 | 90753 | +10818 | 26412 | +12874 |
|  |  | ssp585 | 0.970 | 95904 | +15969 | 14922 | +1384 |
| UKESM1 - 0 - LL | 2071 - 2100 | ssp370 | 0.971 | 96889 | +16954 | 14377 | +839 |
|  |  | ssp585 | 0.973 | 105557 | +25622 | 18684 | +5146 |

Table S2. The contribution of eight non-correlated bioclimatic variables in MAXENT analyses for three models used.

| Code | Bioclimatic variables | Current | Future 1. GFDL-ESM4 | | | | Future 2. MPI-ESM1-2-HR | | | | Future 3. UKESM1-0-LL | | | |
| --- | --- | --- | --- | --- | --- | --- | --- | --- | --- | --- | --- | --- | --- | --- |
|  | Time interval | | 2041 - 2070 | | 2071 - 2100 | | 2041 - 2070 | | 2071 - 2100 | | 2041 - 2070 | | 2071 - 2100 | |
|  | Change scenario | | ssp370 | ssp585 | ssp370 | ssp585 | ssp370 | ssp585 | ssp370 | ssp585 | ssp370 | ssp585 | ssp370 | ssp585 |
| bio1 | Annual Mean Temperature | **20.4** | **19.4** | **20.3** | **19.6** | **18** | **20** | **19.7** | **19.7** | **19.4** | **20.1** | **18.7** | **20.3** | **18.8** |
| bio3 | Isothermality | **10.4** | **11** | **9.8** | **10.2** | **11.5** | **11** | **11** | **10.2** | **10.8** | **9.7** | **10.3** | **11** | **10.9** |
| bio4 | Temperature Seasonality | 1.7 | 1.8 | 1.3 | 1.7 | 2.7 | 2.3 | 2.5 | 2.1 | 1.8 | 2.2 | 2.1 | 2.6 | 2.4 |
| bio8 | Mean Temperature of Wettest Quarter | 3.7 | 4.2 | 2 | 4.3 | 4.5 | 4.3 | 4.5 | 5.1 | 4.4 | 4 | 4.6 | 3.9 | 4.4 |
| bio9 | Mean Temperature of Driest Quarter | 0.7 | 0.7 | 0.4 | 1.4 | 0.5 | 0.9 | 0.7 | 0.8 | 1.0 | 1.1 | 0.9 | 0.7 | 0.8 |
| bio15 | Precipitation Seasonality | 3.4 | 4.6 | 5.5 | 3.8 | 5 | 3.7 | 4 | 3.6 | 3.6 | 3.7 | 4.9 | 3.5 | 4.2 |
| bio18 | Precipitation of Warmest Quarter | **21.9** | **20.7** | **21.2** | **22.2** | **19.8** | **20.3** | **19.1** | **19.3** | **21** | **19.4** | **19.5** | **19.2** | **19.6** |
| bio19 | Precipitation of Coldest Quarter | **37.8** | **37.5** | **39.6** | **36.9** | **38** | **37.6** | **38.5** | **39** | **37.9** | **39.8** | **39** | **38.8** | **38.9** |

Table S3. Values of eight bioclimatic variables for each surveyed population locations from current period (1981-2010) used for PCA.

| **Pop** | **bio1** | **bio3** | **bio4** | **bio8** | **bio9** | **bio15** | **bio18** | **bio19** |
| --- | --- | --- | --- | --- | --- | --- | --- | --- |
| **LC1** | 13.05 | 28.1 | 656.2 | 15.35 | 15.35 | 31.8 | 896.8 | 959.6 |
| **LC2** | 9.05 | 31.0 | 699.6 | 5.95 | 7.55 | 27.6 | 695.4 | 921.9 |
| **LC3** | 11.55 | 32.4 | 730.0 | 7.95 | 10.65 | 24.7 | 358.8 | 501.4 |
| **LC4** | 9.55 | 30.6 | 777.9 | 5.65 | 3.85 | 11.6 | 363.9 | 387.2 |
| **LR1** | 11.95 | 29.7 | 791.4 | 3.45 | 21.35 | 15.0 | 350.1 | 455.9 |
| **LR2** | 8.45 | 29.8 | 794.3 | -0.05 | 18.05 | 18.3 | 234.5 | 332.7 |
| **WGC1** | 11.15 | 28.5 | 793.1 | 2.65 | 20.75 | 13.4 | 276.5 | 333.5 |
| **WGC2** | 10.35 | 29.0 | 787.9 | 6.05 | 4.65 | 10.8 | 302.3 | 320.3 |
| **WGC3** | 10.35 | 29.3 | 786.8 | 6.05 | 19.95 | 12.4 | 327.0 | 372.8 |
| **WGC4** | 11.05 | 30.0 | 773.9 | 2.85 | 20.35 | 12.3 | 454.6 | 531.4 |
| **WGC5** | 11.35 | 29.8 | 769.8 | 3.15 | 20.55 | 13.8 | 439.2 | 538.5 |
| **WGC6** | 9.85 | 29.4 | 780.3 | 5.65 | 4.15 | 9.4 | 662.8 | 590.8 |
| **WGC7** | 12.35 | 30.7 | 747.0 | 21.75 | 2.65 | 14.1 | 857.5 | 616.7 |
| **CGC1** | 9.65 | 28.9 | 864.3 | 13.95 | -1.65 | 33.6 | 240.1 | 120.4 |
| **CGC2** | 11.75 | 28.1 | 857.2 | 15.95 | 0.65 | 37.1 | 307.6 | 132.3 |
| **CGC3** | 11.85 | 28.0 | 853.7 | 16.05 | 0.85 | 36.3 | 296.2 | 131.9 |
| **CGC4** | 11.25 | 26.7 | 831.8 | 15.25 | 0.55 | 36.1 | 356.2 | 151.0 |
| **EGC1** | 9.85 | 27.6 | 838.9 | 13.95 | -0.95 | 40.0 | 446.6 | 156.8 |
| **EGC2** | 11.75 | 28.3 | 841.0 | 16.05 | 0.85 | 37.8 | 434.0 | 166.8 |
| **EGC3** | 10.55 | 26.7 | 861.6 | 14.75 | -0.55 | 32.8 | 284.8 | 126.9 |
| **EGC4** | 8.85 | 26.2 | 823.2 | 12.95 | -1.85 | 27.5 | 235.4 | 138.7 |
